# Model-driven analysis reveals oxidative stress adaptation enabling efficient energy utilization in a Crabtree-negative* Saccharomyces cerevisiae*

**DOI:** 10.1038/s41598-026-58495-x

**Published:** 2026-06-21

**Authors:** Albert Tafur Rangel, Andrés Castillo García, Carl Malina, Verena Siewers, Eduard J. Kerkhoven

**Affiliations:** 1 Department of Life Sciences, Chalmers University of Technology, 41296 Gothenburg, Sweden; 2 Novo Nordisk Foundation Center for Biosustainability, Technical University of Denmark, 2800 Lundtofte, Denmark; 3 Department of Microbiology, Universidad Popular del Cesar, Valledupar, Colombia; 4 SciLifeLab, Chalmers University of Technology, 41296 Gothenburg, Sweden

**Keywords:** Biochemistry, Biological techniques, Biotechnology, Computational biology and bioinformatics, Microbiology

## Abstract

Although abolishing the Crabtree effect in *Saccharomyces cerevisiae* through a pyruvate dehydrogenase bypass eliminates carbon loss through ethanol overflow metabolism, it compromises growth rates. While the Crabtree effect has been a valuable natural adaptation, it is energetically inferior to respiration and is generally undesirable in cell factories engineered to produce assimilatory compounds. Restoring growth efficiency in Crabtree-negative strains remains a central challenge. Through adaptive laboratory evolution of the engineered strain (sZJD23) and subsequent reverse engineering, a variant (sZJD28) with markedly improved growth was identified. This improvement is driven primarily by a mutation in MED2 (encoding a Mediator complex subunit) and, to a lesser extent, a mutation in GPD1 (encoding glycerol-3-phosphate dehydrogenase). By integrating quantitative proteomics with enzyme-constrained genome-scale modelling, we demonstrate that these mutations jointly enable a more efficient mode of oxidative stress adaptation and energy utilization. The GPD1 mutation suppresses a protein-costly, suboptimal NAD⁺-recycling strategy reliant on glycerol synthesis, while the MED2 mutation reshapes the oxidative stress response towards peroxisomal detoxification. Collectively, these adjustments optimize metabolic flux distribution and reduce protein costs in energy metabolism, thereby increasing ATP availability. Our findings reveal how coordinated mutations in regulatory and metabolic genes restore growth fitness in engineered Crabtree-negative yeast.

**Supplementary Information:**

The online version contains supplementary material available at https://doi.org/10.1038/s41598-026-58495-x.

## Introduction

In *Saccharomyces cerevisiae,* adenosine triphosphate (ATP) is primarily regenerated via two metabolic pathways: respiration, which requires oxygen and occurs in mitochondria, and fermentation, which converts glucose into ethanol without oxygen as external electron acceptor.^1,2^ While respiration is more substrate-efficient (higher ATP yield per glucose molecule), fermentation is more protein-efficient (higher ATP production rate per unit of protein).^3^ Interestingly, *S. cerevisiae* can ferment glucose even in the presence of oxygen when sugar is abundant, a phenomenon known as the Crabtree effect.^4^ This effect is not unique to yeast; it is also observed in certain cancer cells (the Warburg effect) and fast-growing bacteria (regarded as overflow metabolism). The Crabtree effect is regulated at multiple levels, including glucose sensing, transcriptional control of glycolytic and respiratory genes, and mitochondrial function. Yeast species that exhibit this behavior are termed 'Crabtree-positive’, whereas those that rely strictly on oxidative respiration are considered 'Crabtree-negative’.

**Fig. 1:**
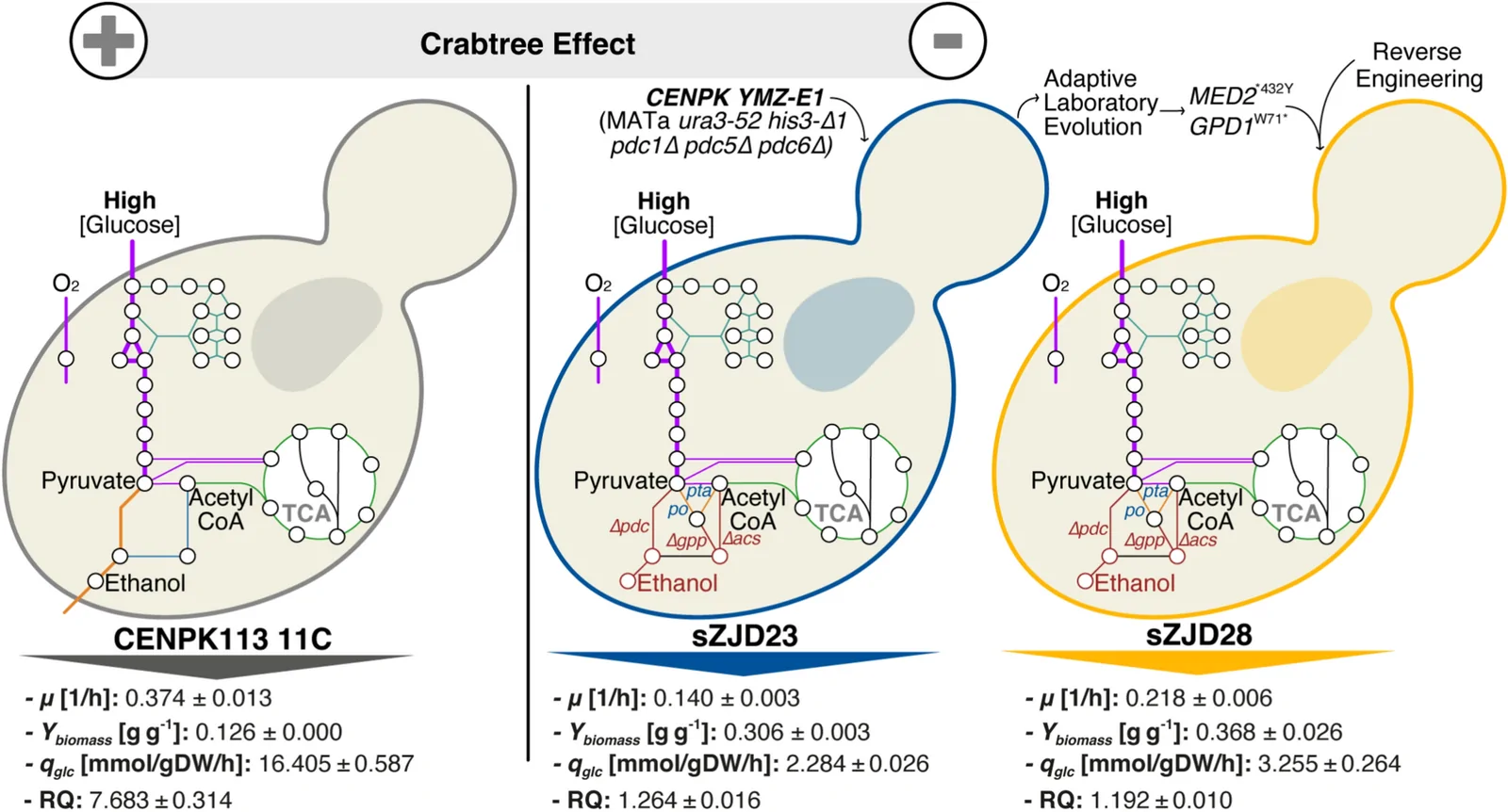
Graphical abstract of results obtained for Dai, Z. et al., and phenotypes of the strain used in this study.^5^

Within their natural sugar-rich ecological niche, the concurrent use of respiration and fermentation confers metabolic flexibility, enabling *S. cerevisiae* to withstand fluctuating environmental conditions. The resulting rapid glucose consumption and ethanol production additionally serve to inhibit competing microorganisms, providing a competitive advantage. In the context of cell factory design, however, this concurrent pathway operation is generally inefficient, as maximizing product yield per unit substrate consumed is a primary engineering objective.^5^ *S. cerevisiae* strains that are deficient in pyruvate decarboxylase (Pdc^–^) do not produce ethanol but exhibit deficient growth in complex medium with glucose and are unable to grow on a defined minimal medium with glucose as the sole carbon source.^6^ A mutation in *MTH1* acquired via adaptive laboratory evolution (ALE) restored growth of the *S. cerevisiae* Pdc^–^ strain in minimal medium; however, growth rates were still low.^7^ To address the growth deficiency with Pdc^–^ strains, a pyruvate dehydrogenase (PDH) bypass was previously introduced yielding *S. cerevisiae* strain sZJD23 (Fig. 1).^5^ This bypass is mediated by pyruvate oxidase (PO*av*, EC 1.2.3.3) from *Aerococcus viridans,* which generates acetyl-phosphate from pyruvate, and phosphotransacetylase (PTA*se*, EC 2.3.1.8) from *Salmonella enterica,* which subsequently converts acetyl-phosphate to acetyl-CoA. Although the integration of this pathway to Pdc^–^ strain improves the growth (0.140 h^−1^), it is still lower than natural Crabtree negative yeast such as *Debaromyces vanrijiae* and *Kluyveromyces aestuarii* (0.347 and 0.429 h^−1^, respectively).^8^ Consequently, the authors performed adaptive laboratory evolution (identifying 18 mutations across 19 genes), and subsequent reverse engineering of the two most dominant mutations: Firstly, a nonstop mutation in *MED2*. Secondly, a nonsense mutation in *GPD1*, which encodes a NADH-dependent glycerol-3-phosphate dehydrogenase. The resulting reverse-engineered sZJD28 strain achieved a growth rate of 0.218 h^−1^ (Fig. 1).^5^

Engineering Pdc^–^ strains to operatve via the PDH bypass demands substantial redistribution in carbon fluxes, which can perturb metabolic homeostasis and elicit stress responses. Transcriptomic analysis of sZJD28 revealed that a nonstop mutation in MED2, a component of the RNA polymerase II Mediator complex, globally reprogrammed gene expression in approximately 33% of the *S. cerevisiae* genome. This simultaneously upregulated translation-associated genes and downregulated glycolytic and carbon metabolism genes, thereby rebalancing the NADH/NAD⁺ ratio and reallocating proteome resources toward ribosome biosynthesis to increase the growth rate of the Crabtree-negative strain.^5^ However, transcript levels alone do not fully capture the functional state of metabolism, as they often do not correlate directly with protein abundance or enzymatic activity, as well as metabolic flux through only an isolated pathway or systemic implications. Moreover, it does not provide a comprehensive explanation as to why a double mutation in *MED2* and *GPD1* is required to achieve the highest growth rate. ^5^ To gain deeper insight into how these evolved strains achieve improved growth, we combined Gene Set Analysis of absolute quantitative proteomics with its integration into an enzyme-constrained model (ecModel) of *S. cerevisiae*. This systems-level approach allows us to quantify and compare metabolic flux distributions in the wild-type and for the primary and reverse-engineered Crabtree-negative strains. Our analysis revealed how evolutionary adaptation reshapes protein allocation and metabolic fluxes across multiple pathways. This points to an alternative strategy for mitigating stress caused by metabolic engineering and improving growth in the absence of the Crabtree effect.

## Results

The physiology of the Crabtree-positive reference strain CEN.PK113-11C and two engineered Crabtree-negative strains, the primary engineered strain sZJD23 and the reverse engineered strain sZJD28, were examined in this study. These three strains were cultured in triplicate in bioreactors. Thereafter, a comparison was made between the physiological parameters obtained in this study and those previously reported. Our data showed that the growth rates of both Crabtree-negative strains were lower (Table 1) than previously reported (Fig. 1). However, compared to sZJD23, the sZJD28 strain still showed a strong improvement in growth rate. This phenomenon was consistent with previous work.^5^ In addition, the CEN.PK113-11C growth rate observed here was higher than that reported previously (Fig. 1 and Table 1). The glucose uptake rate in the wildtype strain was reduced by ca. 12%, implying a higher biomass yield. The reduced glucose uptake rate in the Crabtree-negative strains (both sZJD23 and sZJD28) is coupled with a near-stoichiometric oxygen consumption (RQ ≈ 1), consistent with fully respiratory glucose catabolism, in contrast to the high RQ observed in CEN.PK113-11C, which reflects fermentative activity.

**Table 1:** Physiological parameters of the strains used in this study.

Parameter	CEN.PK113-11C	sZJD23	sZJD28
μ [1/h]	0.419 ± 0.007	0.127 ± 0.006	0.171 ± 0.008
Y_biomass_ [g g^−1^]	0.161 ± 0.012	0.274 ± 0.005	0.376 ± 0.035
*q*_glucose_ [mmol/gDW/h]	14.359 ± 0.894	2.554 ± 0.124	2.526 ± 0.299
*q*_ethanol_ [mmol/gDW/h]	19.420 ± 0.172	0.006 ± 0.007	0.007 ± 0.027
*q*_glycerol_ [mmol/gDW/h]	1.057 ± 0.487	0.001 ± 0.002	0.000 ± 0.000
*q*_acetate_ [mmol/gDW/h]	0.508 ± 0.117	1.063 ± 0.089	0.387 ± 0.183
*q*_pyruvate_ [mmol/gDW/h]	0.100 ± 0.020	0.039 ± 0.007	0.000 ± 0.000
*q*_succinate_ [mmol/gDW/h]	0.008 ± 0.002	0.036 ± 0.005	0.000 ± 0.000
*q*_CO2_ [mmol/gDW/h]	23.493 ± 2.306	5.431 ± 0.260	6.697 ± 0.501
*q*_O2_ [mmol/gDW/h]	5.777 ± 0.633	4.809 ± 0.037	6.323 ± 0.246
RQ	4.066 ± 0.988	1.129 ± 0.013	1.059 ± 0.058

### Absolute quantitative proteomics as a key system differentiator

The regulation of cell growth and by-product generation spans multiple biological layers, from gene expression to metabolism, and disentangling their contributions benefits from single- or multi-omics analyses.^9^ To gain a systemic description of how PDH bypass allows Crabtree-negative strains to increase their growth rate, we performed a proteomics analysis using absolute protein quantifications. A total of 3630 proteins were quantified using tandem mass tag (TMT) based mass spectrometry in triplicate per strain. For this, intensity-based absolute quantification (iBAQ) with proteomics dynamics range standard (UPS2) was used as an internal standard. To minimize noise introduced by low-abundance measurements in downstream differential expression analysis, proteins with normalized abundance values < 0.19 [mg/gDW protein] in at least 6 samples were excluded. This threshold corresponds to the 10th percentile of the normalized abundance distribution. As a result, 3595 filtered proteins were obtained (Supplementary Data 1).

**Fig. 2:**
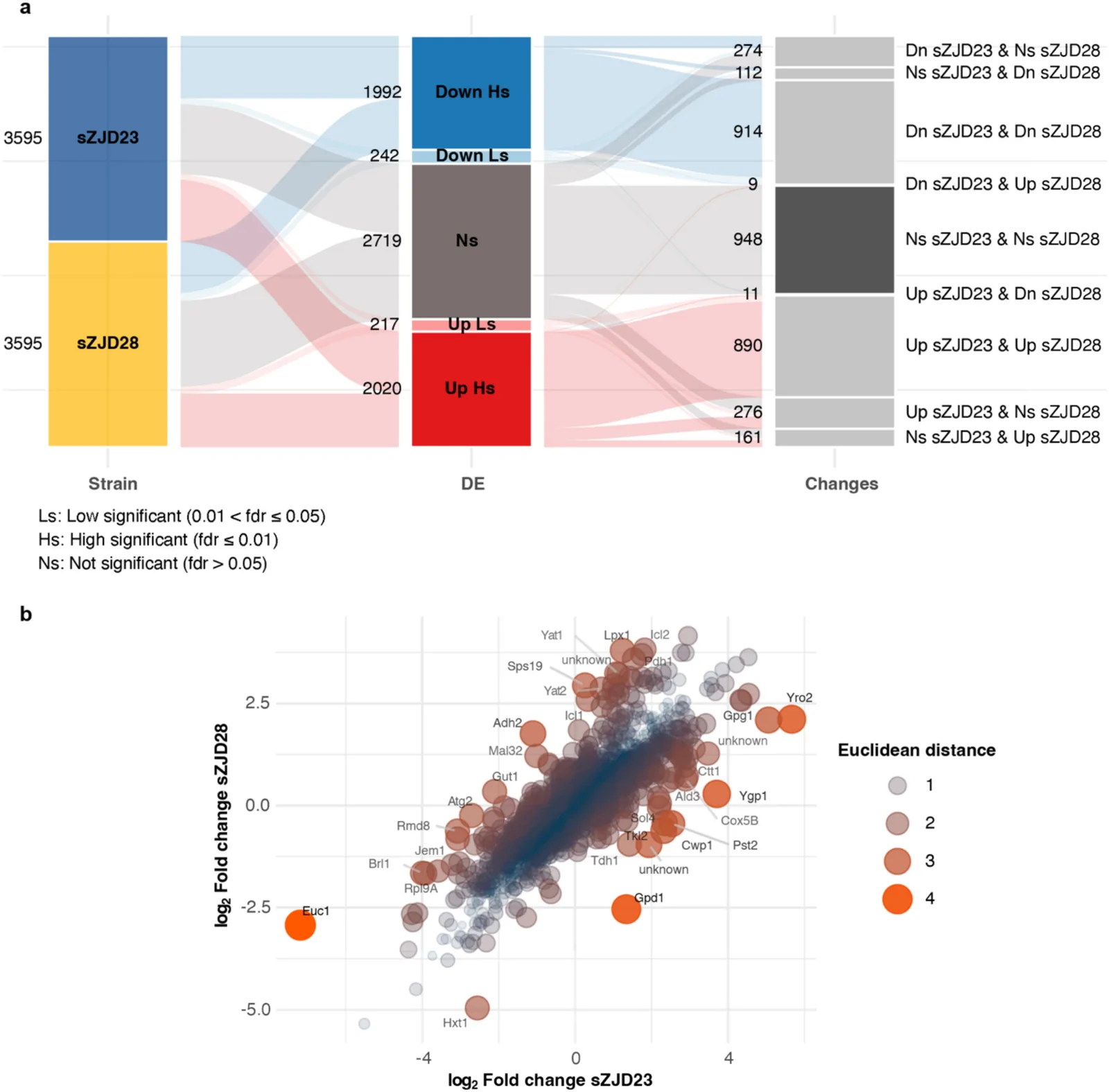
Proteomics analysis. (**a**) Proteins down- and up-regulated in sZJD23 and sZJD28 compared to CEN.PK113-11C. (**b**) Euclidean distance of the log2 fold change of sZJD23 vs. sZJD28.

To characterize differences in protein expression between sZJD23 and sZJD28, a differential expression (DE) analysis was performed using the Crabtree-positive *S. cerevisiae* CENPK113-11C strains as common reference. We highlighted and summarized the DE pattern in Fig. 2a. A protein was defined as DE if the absolute log2 fold change (FC) was > 0 and the false discovery rate (FDR) ≤ 0.05 (marked as low significant in Fig. 2a) or ≤ 0.01 (high significant). Among the more stringently filtered DE proteins, those involved in Glucose transport, the tricarboxylic acid (TCA) cycle, and the respiratory chain were predominant among the more stringently filtered DE proteins. Moreover, it is noteworthy that alcohol dehydrogenases (Adh2/3) enzymes are up-regulated in sZJD28 even though ethanol production is not observed in both sZJD23 and sZJD28.

A comparison of the DE pattern between sZJD23 and sZJD28 reveals nine subgroups, which can be assigned based on the significance and directionality of the expression change (Fig. 2a-changes). Among them, a core of 76.5% (2752 proteins) was identified, following a similar trend in both strains. Of the other 23.5%, 274 and 276 genes were down or upregulated in sZJD23 while non-significant in sZJD28, implying that the mutations in sZJD28 bring it closer to the original reference strain.

Proteomic data revealed that some of genes previously identified by transcriptomics as DE in sZJD23 and sZJD28 showed strain-specific differences in protein abundance relatively to wild-type. For instance, the low-affinity glucose transporter Hxt1 was down-regulated in both data types, but with log2 FC in protein abundance of − 2.56 in sZJD23 and − 4.96 in sZJD28. To better resolve the effects of the PDH bypass and the *MED2* and *GPD1* mutations in Crabtree-negative *S. cerevisiae*. Instead, we compared log2 FC values by computing the Euclidean distance in expression change between sZJD23 and sZJD28 (Fig. 2b), which is equivalent of the absolute log2 fold change between the two strains and is therefore independent of directionality. This identified two groups of differentially expressed proteins:. 621 proteins with a distance of 0.5 to 1, and 214 proteins with distances ≥ of 1 (Supplementary Data 1). The first group was enriched for purine ribonucleotide metabolic process, cellular respiration, carbohydrate metabolic process, beta-glucosidase activity, and oxidoreductase activity. The second was associated with fatty acid beta-oxidation, chaperone cofactor-dependent protein refolding, glycolytic process, energy derivation by oxidation of organic compounds, and oxidoreductase activity, with notable representation of proteins linked to oxidant detoxification, and cytoplasmic stress granule. Peroxisome proteins were also overrepresented among proteins with distance ≥ 1, consistent with their roles in detoxification, lipid metabolism, and hydrogen peroxide metabolism.^10^ Notably, Gpd1 showed the second-highest Euclidean distance (3.87), with reduced expression in sZJD28 relative to sZJD23 (Fig. 2b). GPD1 was previously identified by ALE as a mutation enhancing growth rate in sZJD28.

### PDH bypass induces oxidative stress mediated by pyruvate oxidase.

**Fig. 3:**
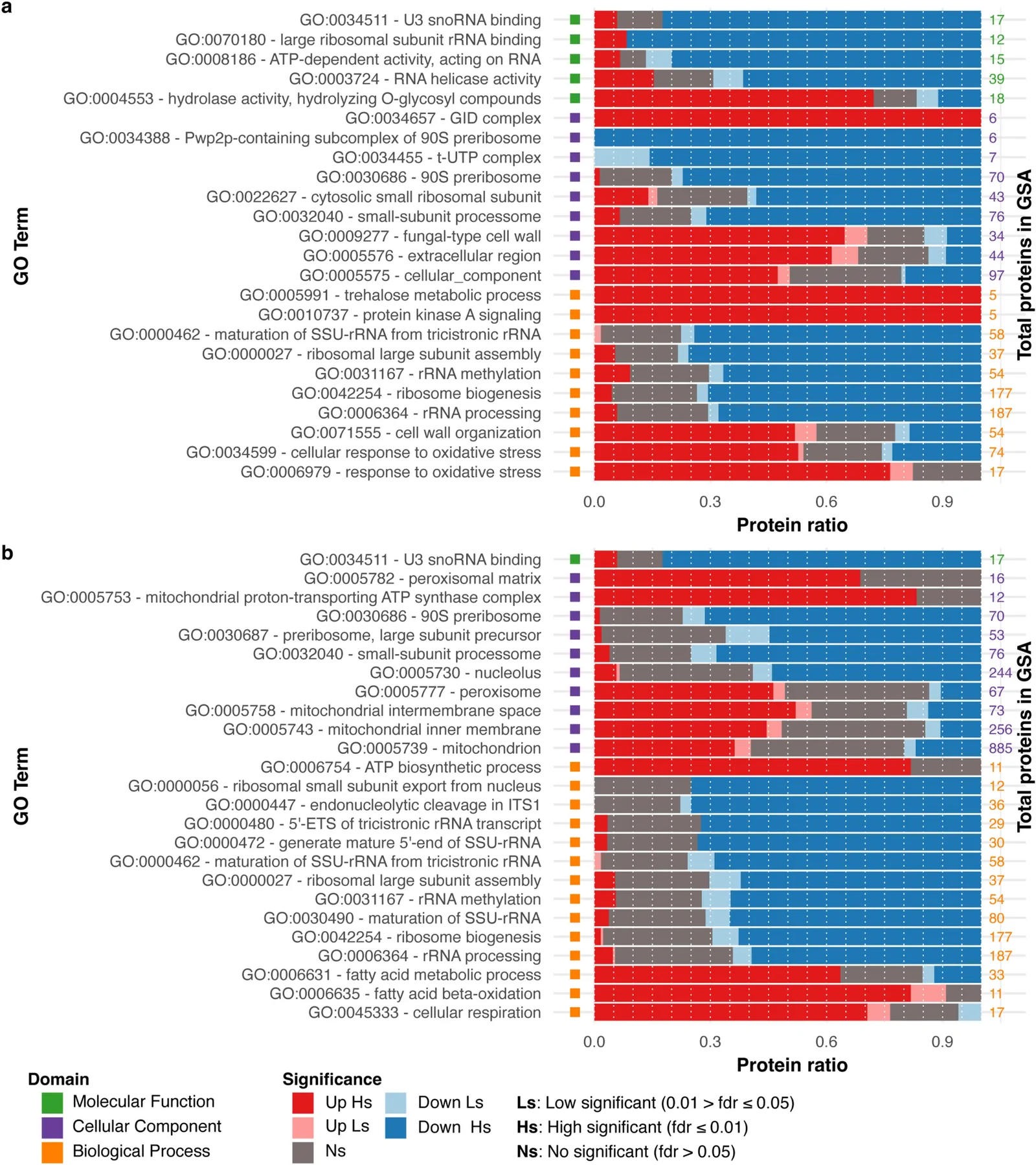
Gene set analysis of proteins differentially expressed in sZJD23 (**a**) and sZJD28 (**b**) compared to CENPK113-11C. The *x-axis* was calculated as the ratio of the number of proteins in the GSA / the total number of *S. cerevisiae* proteins in the GO term. The first 10 lowest values of *p-value* distinct direction down (dist.dir.dn) and the 10 lowest values of *p-value* distinct direction up (dist.dir.dn) are shown. This was done after filtering those GO terms with a non-directional p-value <  = 0.05.

Gene Set Analysis (GSA) of gene ontology (GO) terms identified 46 significantly enrichment terms (non-directional *p* ≤ 0.05) among proteins differentially expressed in sZJD23 and 58 in sZJD28. Figure 3 shows the top ten GO terms by lowest directional *p*-value (up- and down-regulated). Proteins involved in ribosome assembly were downregulated in both strains, consistent with their reduced growth rates, as ribosome production scales with growth.^11,12^ The cells experience oxidative stress, as evidenced by up-regulation of 50% of proteins in the cellular response to oxidative stress GO term (Fig. 3a). This stress is likely driven by the heterologous PO*av*, which generates H_2_O_2_ during pyruvate conversion to acetyl-phosphate.^5^ Superoxide dismutase (Sod1/2), implicated in H_2_O_2_ tolerance^13^, were upregulated in both strains, though oxidoreductase activity (GO:0,016,491) was significantly up-regulated only in sZJD28.

Unlike sZJD23, sZJD28 showed enrichment in peroxisome (GO:0,005,777) and mitochondrion (GO:0,005,739) compartments (Fig. 3b), consistent with transcriptional responses in oxidative-stress-resistant *S. cerevisiae*^14^ , suggesting an ongoing ROS response. Reduced cytosolic catalase Ctt1 (Euclidean 2.20, Fig. 2b) and increased Cta1 in sZJD28 suggest H_2_O_2_ detoxification shifted from cytoplasm to peroxisome. Lower glutathione protein levels further indicate reliance on peroxisomal catalase (Cta1) rather than cytosolic glutathione systems. As Cta1 detoxifies H_2_O_2_ from beta-oxidation^15^, this aligns with the observed upregulation of fatty acid beta-oxidation and metabolism in sZJD28 (Fig. 3b). Notably, thioredoxin reductase Trx2 increased by ca. 1 log2 FC in sZJD28.

In sZJD28, the up-regulation of the mitochondrial ATP synthase complex and respiratory chain complexes (Fig. 3b) suggests an enhanced respiratory and oxidative phosphorylation capacity, likely reflecting a cellular adaptation to meet the elevated ATP demands of oxidative stress defense mechanisms and to maintain redox balance.^15^.

### Model-driven analysis of the Crabtree effect.

Metabolic network modeling is a widely used, computationally based approach to understanding the inner workings of the cell. ^16,17^ However, due to the high complexity and levels of interaction in the cell, traditional approaches such as flux balance analysis (FBA) may predict unrealistic or incorrect inner biological states, such as metabolic fluxes higher than what its enzymes can sustain.^18^ One alternative to make realistic predictions using genome-scale metabolic models (GEMs) is the integration of enzymatic constraints and proteomics data.^18–21^ Here we predicted internal fluxes for the three strains by applying the GECKO approach that considers enzyme kinetics through *k*_cat_ values and protein abundances through absolute concentrations [mg/gDW].^2,19,22^ As a result of the yeast-GEM expansion to an enzyme-constrained model (ecModel), a total of 1156 proteins were incorporated in the model, and the initial total cellular protein content was assumed to be 0.5 [g/gDW], before strain-specific protein levels were integrated below.

**Fig. 4:**
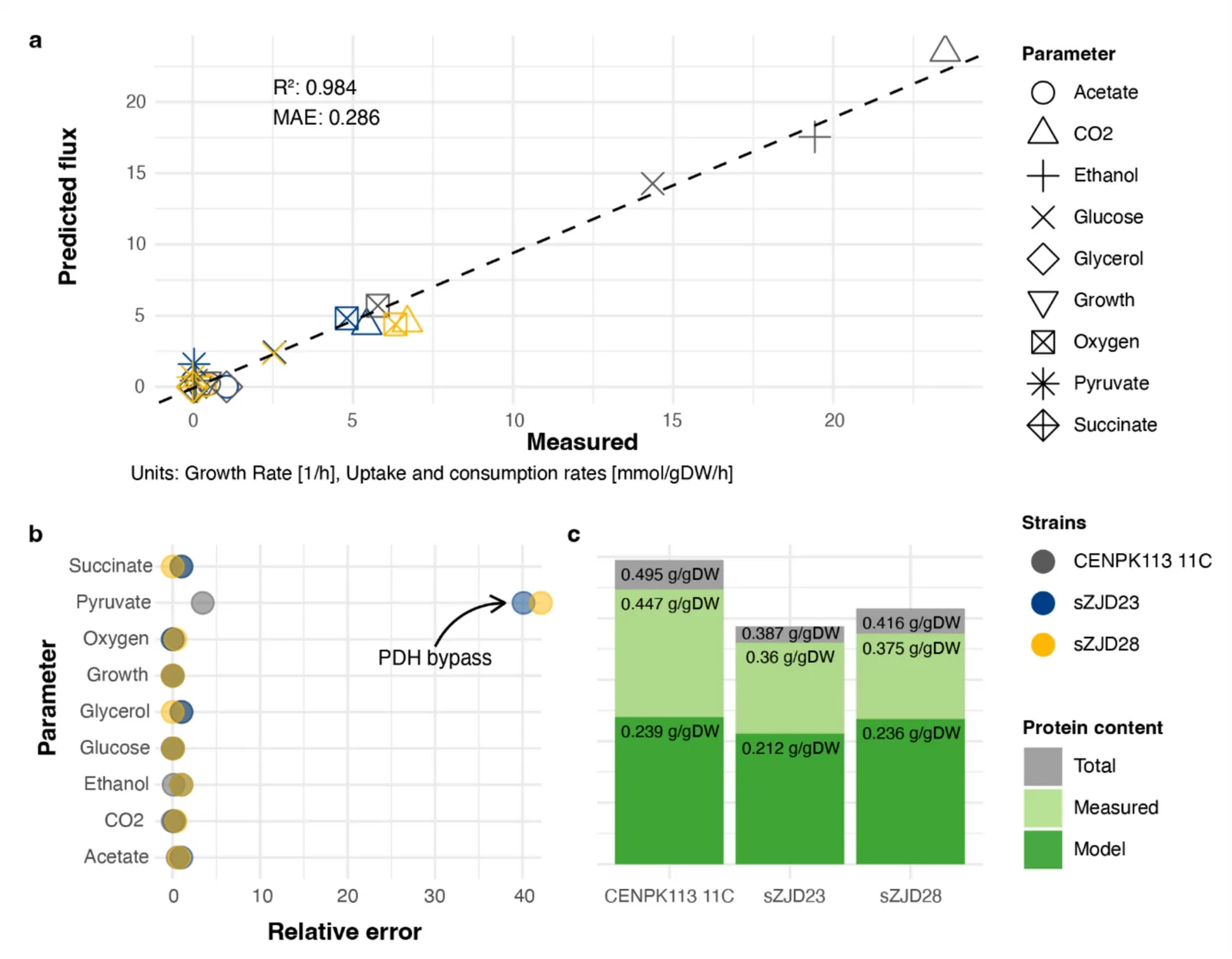
Metabolic flux distribution of CEN.PK113-11C, sZJD23, and sZJD28. (**a**) The strain-specific proteomics-integrated models predicted fluxes (by pFBA) that are close to the experimentally measured values. (**b**) Individual relative error of each experimental parameter measured and used during proteomics integration. (**c**) Protein content per strain, total corresponds to the total protein content in the cell as measured from cell extracts by Branford assay, measured corresponds to the sum of all protein levels measured by absolute proteomics, and model corresponds to the sum of all constrained proteins with measured protein concentrations.

The main difference between CEN.PK113-11C and sZJD23, and sZJD28 is the presence of the Crabtree effect. To increase the confidence of this model-driven analysis, it was first assessed whether the ecModel could replicate the Crabtree effect by comparing the FBA-predicted and reported measured exchange rates in *S. cerevisiae*,^23^ which yielded a successful result (Supplementary Fig. 1). Then, the resulting ecModel was used to build condition/strain-dependent proteome models by incorporating experimentally measured growth rates, exchange uptake/production rates, and proteomic constraints (protein concentration [mg/gDW]). Figure 4a shows the growth rate and exchange rates predicted by performing a parsimonious FBA (pFBA) using the proteome models. Simulations were performed by minimizing glucose uptake rate in absence of non-proteomics constraints and fixing the specific growth rate to its maximum attainable value. The phenotype predicted in each strain-specific model shows high accuracy, although the sZJD23 and sZJD28 proteome models predicted pyruvate overflow, which was not observed experimentally (Fig. 4b).

After integration of proteomics data in the ecModels it appeared the weight-fraction of modelled protein was very similar across all three models (variance was 0.000219 [g/gDW]), even though the total protein content and the proteomics-measured protein content differed by ca. 0.1 g/gDW between CEN.PK113-11C and the Crabtree-negative strains (Fig. 4c). The lower protein weight-fraction in the Crabtree-negative strains sZJD23 and sZJD28 is mostly a consequence of differential expression of non-metabolic genes.

To understand the metabolic alterations between strains, fluxes of all the reactions were normalized by the growth rate, and protein usage fluxes were summed based on their assignment to KEGG pathways (Supplementary Fig. 2). Interestingly, it was observed that even while the median protein levels in carbohydrate metabolism were very similar across all strains, sZJD28 has a relatively low flux through energy metabolism pathways compared to CEN.PK113-11C and sZJD23. sZJD28 appears to have evolved to be more energetically efficient, as model simulations determine ATP turnover per glucose consumed at 4.28, 6.46 and 7.73 mol/mol for CEN.PK113-11C, sZJD23 and sZJD28, respectively. This agrees with the observations from natural Crabtree-negative microorganisms.^2^ When *S. cerevisiae* catabolizes glucose, the carbon flux will go through central carbon metabolism, but the level of this flux will depend on metabolite concentrations and enzymatic activities. Initially, glucose is phosphorylated by hexokinases to produce glucose 6-phosphate. Subsequently, it is metabolized via either glycolysis, mainly used for glucose degradation, producing ATP and NADH. Alternatively, it is metabolized via the Pentose Phosphate Pathway (PPP), which produces NADPH through its oxidative branch, which is vital to handle oxidative stress.^24^ The split ratio (glycolysis/PPP) observed in these pathways was 3.25, 12, and 4.25, for CENPK113-11C, sZJD23, and sZJD28, respectively. A higher relative flux going through the PPP in sZJD28 compared to sZJD23 may indicate why the oxidative stress GO term is not enriched in this strain (cf. Fig. 3), as the increased NADPH production used as cofactor by enzymes would have aided the oxidative stress response.^24^.

Glucose metabolized through glycolysis can be used to produce glycerol by conversion of dihydroxyacetone phosphate to glycerol-3-phosphate (G3P) by NAD-dependent glycerol 3-phosphate dehydrogenase (Gpd1/2) and subsequent conversion of G3P to glycerol by the glycerol 3-phosphate phosphatases (Gpp1/2). In sZJD23, *GPP1* and *GPP2* were deleted to avoid the conversion of acetyl-phosphate to acetate^5^, which can result in an increased level of G3P (Fig. 1) and reduced glycerol production (Table 1). *S. cerevisiae* that lacks both glycerol 3-phosphatases is hypersensitive to oxidative stress when exposed to H_2_O_2_, and these cells redirect their carbon flux from glycolysis to the PPP in favor of NADPH regeneration.^25–27^ Although CEN.PK113-11C generated glycerol in our experiments; the proteome model without any by-product constraint did not predict any glycerol production. This was even though the model predicted a flux through the glycerol 3-phosphate dehydrogenase reaction. Still, this was rather directed towards other pathways, e.g., lipid biosynthesis. ^28^ The glycerol 3-phosphate dehydrogenase fluxes were 0.081, 0.168, and 0.21 mmol/gDW/h (normalized values) in CEN.PK113-11C, sZJD23, and sZJD28, respectively. Meanwhile, in sZJD28, FAD-dependent glycerol-3-phosphate dehydrogenase Gut2, which converts G3P to dihydroxyacetone phosphate, had an increased flux compared to sZJD23 and CEN.PK113-11C, suggesting this reverse pathway as the way to handle G3P accumulation and subsequently re-routing the flux through glycolysis while helping in oxidative stress ^14,29–31^ in favor of NADPH regeneration.

**Fig. 5:**
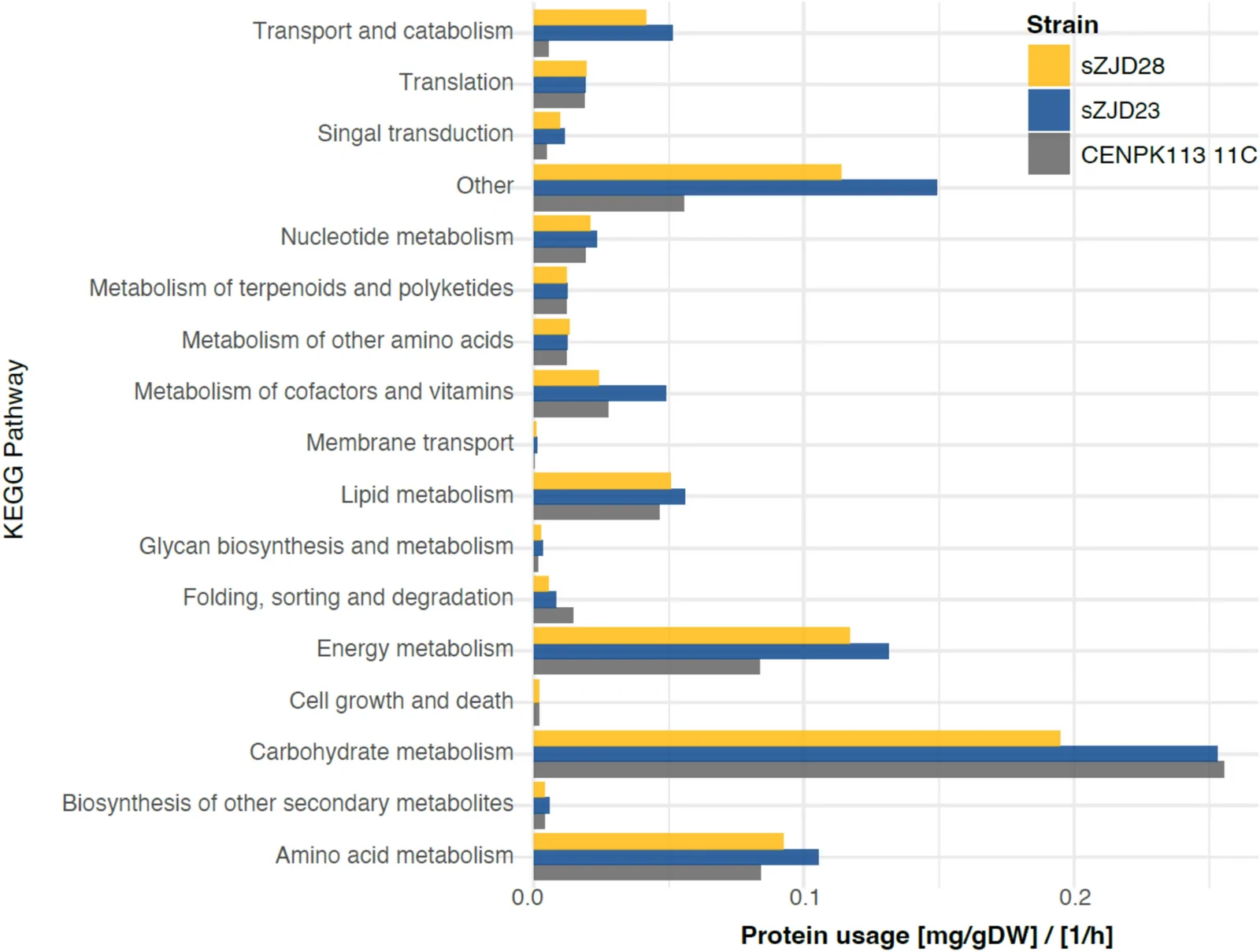
Normalized protein usage per KEGG pathway.

It has previously been described that the full oxidation of glucose is costly in terms of protein, contrasting to the lower protein cost of glucose fermentation to ethanol.^2^ This behavior is illustrated in Fig. 5, where both sZJD23 and sZJD28 had higher protein usage requirements [g/gDW] in relation to energy metabolism, whilesZJD28 has evolved lower requirement for proteins involved in energy metabolism.^23,32^ Protein usage is the amount of protein that the model predicts as required to enable the predicted metabolic fluxes. The energy metabolism network consists of glycolysis, the TCA cycle, and oxidative phosphorylation. Our results showed that protein usage related to carbohydrate metabolism is very similar between CEN.PK113-11C and sZJD23, but with a reduction of around 0.05 g/gDW in the protein usage of this pathway in sZJD28. When observing this KEGG pathway in more detail (Supplementary Table 1), the summed usage of glycolysis proteins is 0.0981 [g/gDW] in CEN.PK113-11C, 0.0717 g/gDW in sZJD23, and 0.0425 g/gDW in sZJD28. In the TCA cycle, the summed protein usages of sZJD28 (0.0250 g/gDW) and sZJD23 (0.0351 g/gDW) were increased when compared to CEN.PK113-11C (0.0088 g/gDW). Finally, similar to the TCA cycle, oxidative phosphorylation has the lowest usage in CEN.PK113-11C, and the highest in sZJD23, with a reduction of 0.01 g/gDW in sZJD28. Taken together, these findings suggest that sZJD28 may use carbon for growth and exhibit a higher utilization of the PDH bypass.

To test the hypothesis that sZJD28 might be better adapted to handle oxidative stress, as an explanation for its higher growth rate, we compared sZJD23 and sZJD28 on agar plates at different H_2_O_2_ concentrations (Supplementary Fig. 4). Comparisons across strains were somewhat complicated by the difference in growth rates among the strains. Therefore, the no-H_2_O_2_ control plates were used to select time points for comparison across strains, and the effect of H_2_O_2_ at those time points was assessed. Growth was not strongly influenced until the H_2_O_2_ concentration reached 0.6 mM, at which point the effect on sZJD23 was more pronounced than on sZJD28 and CEN.PK 113-11C, indicating a higher oxidative stress sensitivity of this strain (Supplementary Fig. 4).

**Fig. 6:**
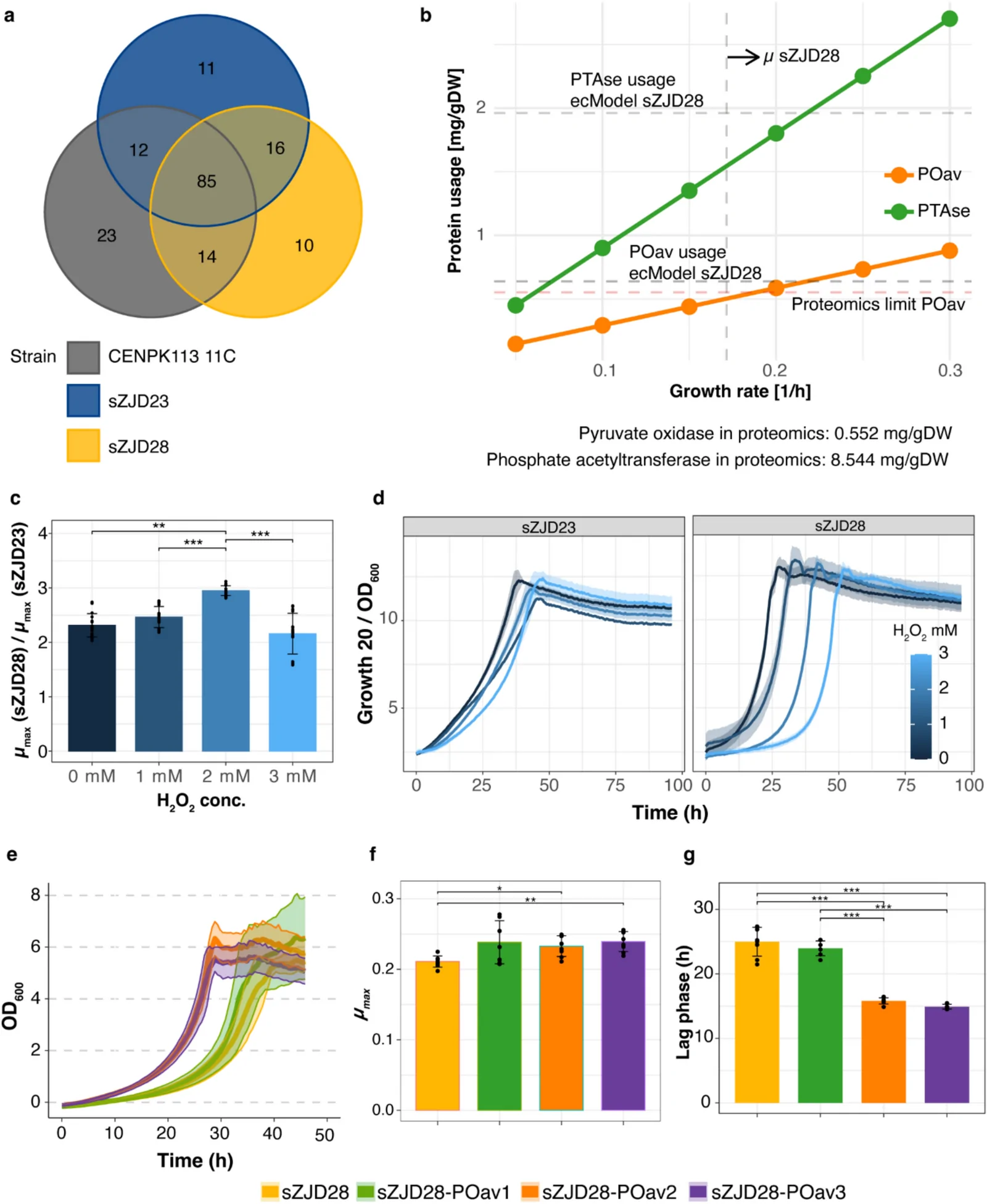
Identification of potential growth-limiting proteins in sZJD28 and comparison of oxidative stress tolerance between sZJD23 and sZJD28. (**a**) Venn diagram of proteins with a capacity usage of more than 0.75. (**b**) Protein usage requirements of PDH bypass proteins in sZJD28. (**c**) Comparison of *μ*_*max*_ ratio between sZJD28 and sZJD23 growth in liquid cultures under different concentrations of H_2_O_2_. Results shown are the average ± SD values for all the possible ratios of *μ*_max_ across strain replicates within each culture. (**d**) Growth curves of sZJD28 and sZJD23 in liquid cultures under different concentrations of H_2_O_2_. *: p < 0.05; **: p < 0.01; ***: p < 0.001. (**e**)**.** Growth curves of sZJD28 and three independent transformants carrying a second copy of PO*av*. Growth was assessed in Delft minimal medium using a GrowthProfiler. Biological and technical triplicates were carried out. Shading illustrates standard deviation. (**f**)**.** Comparison of *µ*_max_ between sZJD28 and the clones carrying a second copy of PO*av*. Results shown are average ± SD values of *µ*_max_ of all the replicates per strain. (**g**)**.** Comparison of lag phase length between sZJD28 and the clones carrying a second copy of PO*av*. Results shown are average ± SD values of the lag phase of all the replicates per strain.

In addition, the strains were cultivated in liquid media to evaluate their growth rates and lag phases. Here, the lag phase of sZJD28 increased in duration with higher H_2_O_2_ levels, while the lag phase of sZJD23 remained largely unaffected (Fig. 6d). Contrastingly, when comparing the *µ*_*max*_ ratios (*µ*_*max*_ sZJD28 / *µ*_*max*_ sZJD23) at different H_2_O_2_ concentrations (Fig. 6c), it appeared that this ratio increased at lower concentrations of H_2_O_2_ (up to 2 mM). Beyond this, the ratio returned to a non-statistically significant difference compared to the ratio at 0 mM H_2_O_2_ (albeit this observation might also be explained by the lower reproducibility of the growth rate measurements of sZJD23 at 3 mM (Supplementary Fig. 3)). The change in ratio at moderate H_2_O_2_ levels was mainly due to the reduced growth rate of sZJD23.

### Growth-limiting proteins in Crabtree-negative *S. cerevisiae*

To investigate how to improve the growth rate in Crabtree-negative strain sZJD28, we searched for limiting proteins, i.e. proteins operating near their measured capacity. Limiting proteins were defined as those for which the required usage in the proteome model simulations exceeded a conservative 75% of the measured protein concentration. According to this definition, 171 proteins were identified as limiting in at least one strain (Fig. 6a). Almost half of these proteins were identified as limiting in all three strains, making them potential targets for metabolic engineering to improve the growth of sZJD28. Curiously, pyruvate oxidase was identified as the unique limiting protein in sZJD28. To further investigate this, we simulated the sZJD28 ecModel without proteomics constraints and various growth rates, while monitoring the required usage of the PTA*se* and PO*av* enzymes (Fig. 6b). As anticipated, the usages of PTA*se* and PO*av* scale with the growth rate. When compared to their measured levels from proteomics, there should be ample PTA*se* to theoretically support growth of more than 0.3 h^-1^, while the measured PO*av* level would not suffice for growth far over 0.2 h^-1^. Indeed, the maximum growth rate of sZJD28 was 0.218 h^-1^, as shown in Fig. 1. This suggests that the level of PO*av* (and not PTA*se*) is potentially limiting during growth.

Finally, to test the hypothesis that increasing PO*av* levels might increase the growth rate of sZJD28, a second copy of the PO*av* expression cassette was integrated into the strain sZJD28. Three independent transformants were isolated for further analysis: sZJD28-POav1, sZJD28-POav2, and sZJD28-POav3. Although variation was found among biological replicates of each colony when measuring growth, we observed a clearly shortened lag phase in two out of the three transformants (Fig. 6e,g). Clone sZJD28-POav1 showed the largest variation among biological replicates compared to the other two clones. In addition, there were no statistical differences in the lag phase of sZJD28-POav1 with sZJD28. A potential explanation for the differences between sZJD28-POav1 and the other two clones may have been the emergence of undesired genetic alterations during the transformation, which, in fact, presented some difficulties during the experimental process.

In addition, significant differences between the maximum growth rates were found between sZJD28 and the clones sZJD28-POav2 and sZJD28-POav3 (Fig. 6f). The high variation among biological replicates of the clone sZJD28-POav1 may lead to reduced statistical power, which could explain the non-significant differences with sZJD28. The introduction of a second copy of PO in sZJD28 led to a 12,9% increase in average µmax for sZJD28-POav1, 10,37% for sZJD28-POav2, and 13,39% for sZJD28-POav3 in comparison with sZJD28.

## Discussion

Cells may respond to environmental changes or disturbances caused as a side effect of metabolic engineering, e.g., various forms of regulation and/or metabolic alterations. Previously, the introduction of a double mutation in *GPD1*^W71^^*^ and MED2^*432Y^ into a Crabtree-negative *S. cerevisiae* strain resulted in altered expression of TATA-containing genes related to cellular amino acid metabolic processes, carbon metabolism, and chemical oxidative stress.^5^ The alteration of the Mediator complex due to the *MED2* mutation was proposed as the source of global rewiring of central metabolism to support faster growth of the Crabtree-negative strain*.* Nevertheless, in this study, we propose that alteration of *GPD1* also plays a significant role in this global rewiring as a response to oxidative stress. Our findings support why individual mutants of *GPD1*^W71*^ and MED2^*432Y^ have lower growth rates (0.139 and 0.156 1/h, respectively)^5^ than the double mutant (0.218 1/h—Fig. 1), as central metabolism and oxidative stress rewiring are complementary in improving the phenotype of Crabtree-negative *S. cerevisiae*.

Alterations in carbohydrate metabolism are essential for protection against hydrogen peroxide.^26,33,34^ Gpd1 is a key NAD^+^-dependent enzyme of glycerol synthesis, consuming up to 5% of the carbon source^35^ and, under aerobic conditions, it is part of a shuttle to maintain intracellular redox balance via glycerol 3-phosphate (G3P).^36^ One strategy by which *S. cerevisiae* deals with oxidative stress is to increase *GPD1* expression, although the underlying mechanism is not clear.^26,37^ Proteomics results suggest that sZJD23 experiences higher oxidative stress, likely due to H_2_O_2_ generated by the heterologous enzyme PO*av*, and further aggravated by the knockout of *GPP1*/*2*. At the same time, an increased flux through the PPP has been observed,^31,33^ generating the required NADPH to keep glutathione reduced. *GPD1* upregulation in proteomics and increased flux through the PPP in FBA simulations were also observed in sZJD23.

While these metabolic alterations in sZJD23 likely reflect a cellular response to oxidative stress, their impact on stress tolerance remains unclear. These alterations may have been a contributor to the observed lower growth rate, although a direct causal relationship has not been established. ALE allowed the cells to find an alternative way to respond to oxidative stress and improve the growth rate of sZJD23. From this, two mutations were reverse engineered to form sZJD28, which has a higher growth rate than sZJD23, suggesting that these mutations working together have indeed helped the cell. Thus, *GPD1* also appears to play a significant role in this global rewiring. Reverse engineering of *GPD1* in position W71 introduced a stop codon, which results in a truncated and most likely non-functional protein. Previous studies reported that the activity of the FAD-dependent Gut2 enzyme was higher in a *gpd1*Δ background compared to wild-type strains, indicating a potential regulatory role of Gpd1 on Gut2.^36^ Our results corroborate these findings, as Gut2 had higher abundance and usage in sZJD28 compared to both CEN.PK113-11C and sZJD23 after modeling proteomics integration for each strain (Supplementary Data 1 and Supplementary Table 1).

Exposure to H_2_O_2_ can result in dramatic cellular changes that affect metabolism, upon which cells reroute their metabolic flux. Carbohydrate fluxes are redirected to the regeneration of NADPH via PPP at the expense of glycolysis in wild-type strains.^26^ However, studies have reported that at high H_2_O_2_ concentrations, glycolysis is energetically more favorable for cellular respiration.^31^ The PPP plays a crucial role in redox balancing during oxidative stress by maintaining high NADPH/NADP + ratios in the cell. At high H_2_O_2_, cells can redirect the glycolytic flux towards the PPP, providing sufficient NADPH to regenerate the glutathione involved in detoxifying H_2_O_2_. Glutathione reductase and thioredoxin reductase, using FAD instead of NADPH as cofactor, are an alternative pathway to convert hydrogen peroxide to water.^34^ A major rerouting of the metabolic flux from glycolysis to PPP was observed in sZJD23 compared to CEN.PK113-11C and sZJD28, while also in sZJD28, the split ratio was still higher than in CEN.PK113-11C. The oxidative stress can be behind the increase in PPP utilization in sZJD23, although this results in the cell increasing the ATP production at the expense of high protein cost (Fig. 6).^23,31,33^ When using the G3P shuttle, the respiratory activity is lower but offers a higher P/O ratio compared to external NADH dehydrogenase. However, the external NADH dehydrogenase has a higher capacity to produce ATP per unit time compared to the shuttle.^36^.

We propose that the *GPD1* mutation in sZJD28 counteracted the suboptimal strategy of *GPD1* upregulation that sZJD23 followed as a “quick” response to increased oxidative stress. Increased NADPH generation via the PPP and a *GPD1*, *GPP1*/*2,* and *GCY1* upregulation would have formed another loop to generate more NADPH and maintain redox balance. However, it is a strategy that decreases cell growth, probably because the G3P shuttle becomes progressively less important when high rates of ATP are needed.^36^ In sZJD23, this would likely have increased G3P. In sZJD28, G3P might not accumulate to the same extent due to the inactivity of Gpd1, while G3P can still be produced by Gpd2 if intracellular NADH accumulates. ^28,31^ Switching off GPD1, introducing a nonsense mutation would then at least disable this ineffective strategy of increased PPP and G3P shuttle, slightly increase glycolytic flux, and ultimately higher production of acetyl-CoA for higher growth rates. Nevertheless, dealing with oxidative stress would still be an issue. In fact, increased POav usage would generate more H_2_O_2_, hindering growth when compared to wildtype strains.

The results obtained from the growth of both strains in solid and liquid media supplemented with H_2_O_2_ support this theory, as the growth rate of sZJD28 is less affected by increasing H_2_O_2_ concentration than the growth rate of sZJD23. Nevertheless, it is interesting to note that the length of the lag phase increased for sZJD28 as the concentration of H_2_O_2_ was increased. This may be due to the intracellular location of the stress response mechanisms of both strains. While in sZJD23, the oxidative stress response includes mainly cytoplasmic proteins, in sZJD28 is more compartmentalized. While sZJD28 might more efficiently deal with the intrinsic oxidative stress due to the PDH bypass in comparison to sZJD23, it appears to render it less well-suited to deal with additional oxidative stress. Contrastingly, the higher availability of ATP in sZJD28 due to the up-regulation of the mitochondrial synthase complex and the mitochondrial respiratory chain complex III may be a key factor in the higher *µ*_*max*_ of sZJD28 under oxidative stress.

The introduction of a second copy of POav in the strain sZJD28 leads to an increase in its µmax, which aligns with the theory that POav abundance is, in fact, limiting growth. In addition, a clear reduction of the lag phase can be observed in at least some of the strains carrying a second copy of POav. It is possible that sZJD28, with a single copy of POav, suffers from a bottleneck in acetyl-CoA generation, which leads to the predicted and observed limitation in µmax compared to the strains with a second copy of POav. This bottleneck could also be responsible for the elongated lag phase in sZJD28, since the lag phase acetyl-CoA availability is of key importance due to the elevated metabolic activity that precedes exponential growth.

The increased growth rate and the tendency towards a shorter lag phase are of interest, not only because of the potential acceleration of the growth process of this Crabtree-negative strain, but also because of the value of acetyl-CoA as a precursor of many industrially relevant products.

## Methods

### Culture conditions

In this study, three *S. cerevisiae* strains were used: CENPK113-11C (MATa MAL2-8c SUC2 *his3*Δ1 ura3-52), sZJD23 (*MAT*a *ura3-52 his3*-∆*1 pdc1*∆ *pdc5*∆ *pdc6*∆ *acs2*Δ::*TEF1*p-POav-*ADH1*t *acs1*Δ::*TEF1*p-PTAse-*CYC1*t *gpp1*Δ *gpp2*Δ), and sZJD28 (sZJD23, MED2^*432Y^, *GPD1*^W71*^). Batch cultures were carried out using Delft minimal medium (2% glucose, 5 g/L (NH_4_)_2_SO_4_, 3 g/L KH_2_PO_4_, 0.5 g/L MgSO_4_⋅7H_2_O, 1 mL/L trace metal solution, and 1 mL/L vitamin solution) supplemented with histidine and uracil in DASGIP 1-L bioreactors with a working volume of 700 mL at 30°C and pH control. The trace metal solution consisted of 19 g/L Na_2_EDTA⋅2H_2_O (disodium ethylenediaminetetraacetate dihydrate), 4.5 g/L ZnSO_4_⋅7H_2_O, 1 g/L MnCl_2_⋅4H_2_O, 0.3 g/L CoCl_2_⋅6H_2_O, 0.3 g/L CuSO_4_⋅5H_2_O, 0.4 g/L Na_2_MoO_4_⋅2H_2_O, 4.5 g/L CaCl_2_⋅2H_2_O, 3 g/L FeSO_4_⋅7H_2_O, 1 g/L H_3_BO_3_, and 0.1 g/L potassium iodide. The vitamin solution consisted of 0.05 g/L d-biotin, 0.2 g/L 4-aminobenzoic acid, 1g/L nicotinic acid, 1 g/L D-pantothenic acid hemicalcium salt, 1 g/L pyridoxine–HCl, 1 g/L thiamine-HCl, and 25 g/L myo-inositol.

### Analytical methods and proteomics analysis

All the analyses were carried out based on three biological replicates. Determination of dry cell weight was performed by filtering the samples using pre-weighed filters (0.45 mm) and subsequently microwaving at 125 W for 15 min. Then, the filters were stored in a desiccator for at least 3 days. For quantification of glucose and by-products (glycerol, acetate, ethanol, pyruvate, and succinate), high-performance liquid chromatography was used, equipped with an Aminex HPX-87H ion-exchange column (Bio-Rad Laboratories, USA) operated at 45°C, a flow rate of 0.6 mL·min^−1^ using 5 mM H_2_SO_4_ as the mobile phase. Samples were previously filtered through 0.45 mm nylon filters. Oxygen and carbon dioxide concentrations were measured in real-time using a GA4 gas analyzer. OUR and CER were calculated as reported by Malina et al*.*^2^

Proteome measurements were carried out following the protocol described by Malina et al*.*^2^ Differentially expressed proteins were determined in R version 4.1.2^38^ using the Limma package.^39^ gene set analysis was carried out using the Piano package^40^ and the mean method. log2 fold change and FDR were used as input, and the Ensembl gene set GO terms database for *S. cerevisiae was* obtained using biomaRt^41,42^. Visualization and data manipulation were made using tidyverse packages.^43–47^.

### Proteomics integration and enzyme-constraint modeling

The yeast consensus genome-scale model v8.6.2 ^48,49^ was used in this study. The model was set up with the PDH bypass by incorporation of two reactions: Pyruvate oxidase (1) and Phosphate acetyltransferase (2).

1 $$pyruvate + phosphate + {\mathrm{O}}_{2} \stackrel{\mathrm{D}4\mathrm{Y}\mathrm{I}\mathrm{P}5}{\to } acetyl-phosphate + {\mathrm{C}\mathrm{O}}_{2} + 2 {\mathrm{H}}_{2}{\mathrm{O}}_{2}$$

2 $$acetyl-phosphate + coenzyme A \stackrel{\mathrm{Q}8\mathrm{Z}\mathrm{N}\mathrm{D}6}{\to } acetyl-CoA + phosphate$$

Bounds of PDH bypass reactions were set to 1e-5 to avoid potential use of these reactions during model expansion and *k*_*cat*_ tuning. The yeast-GEM was expanded to an ecModel by using GECKO v3.0.2^22^ and only using Enzyme Commission Number (EC Number) obtained from UniProt database.^50^ *k*_*cat*_ values obtained from both DLKcat and Brenda were allowed for automatic constraints, although, in some cases, custom *k*_*cat*_ values (with values reported in both databases) were used.^51,52^ The resulting model was compared to measured data to test if the model could predict the Crabtree effect by running FBA at several fixed growth rates and glucose minimization.

Proteomics integration was carried out by setting the upper bound of PDH bypass reactions to 1000. Then, condition/strain-specific models were created. In CEN.PK113-11C model, protein usage reaction of D4YIP5 and Q8ZND6 were set to zero, since those proteins are not present in this strain. For the metabolically engineered strains (sZJD23 and sZJD28), protein usage of Pdc1, Pdc5, Pdc6, Gpp1, Gpp2, Acs1, and Acs2 were set to zero. Into each condition-specific model, experimentally measured protein content, glucose uptake rate, by-product production rates, and absolute protein abundances [mg/gDW] were incorporated. MATLAB R2022a with Gurobi 9.5.1 as solver in the RAVEN toolbox^53^ was used for simulations. Visualization and data manipulation of the results were made using tidyverse packages.^43–47^.

### Strain construction

For the introduction of a second copy of pyruvate oxidase in the sZJD28 strain, PCRs were carried out. Primer sequences are shown in Supplementary Table 4. A fragment consisting of the *TEF1* promoter PO*av* gene from the strain sZJD28, a fragment consisting of the *ADH1* terminator from the plasmid pYTK053^54^, and two fragments corresponding to the X-3 up and down recombination sequences from the plasmid pMC-X3^55^ were amplified with the adequate overhangs to further carry out a fusion PCR. The resulting DNA fragment consisted of *TEF1* promoter, PO gene, and *ADH1* terminator, flanked by the X-3 up and X-3 down sequences form homologous recombination with the integration site X-3, validated for expression of heterologous genes in *S. cerevisiae*^56^. After the fusion PCR, the fragment was purified by gel electrophoresis and by using GeneJET Gel Extraction Kit (ThermoFischer) and used as a repair fragment in the yeast transformation. The sZJD-28 strain transformation was done using the LiAc method, using the assembled repair fragment and the plasmid pMEL-10^57^ encoding a gRNA targeting the established X-3 integration site. After the transformation, colonies were grown in SD-URA agar plates, and the integration of the POav cassette was checked by PCR.

### Growth measurements

Three independent clones of the sZJD28 transformed with a second copy of POav were used for the growth analysis: sZJD28-POav1, sZJD28-POav2, and sZJD28-POav3, including also the parental strain sZJD28. Three pre-cultures per strain were grown to the exponential phase, each one of them from different clones. These biological replicates were then inoculated in technical triplicates in a final volume of 250 μL of Delft liquid media, in a 96-well plate (Enzyscreen CR1496d) and grown in a Growth Profiler 960 (Enzyscreen) at 30°C, 250 RPM. Data were collected for 40 to 50 h and analyzed using the growth rates package in R. Delft liquid medium consisted of 2% glucose, 5 g/L (NH4)2SO4, 14.4 g/L KH2PO4, 0.5 g/L MgSO4⋅7H2O, 150 mg/L uracil, and adjusted to pH 6.5, 1 mL/L trace metal solution, and 1 mL/L vitamin solution.

The average μmax of each strain was obtained from the μmax of each strain replicate. Due to the lack of homogeneity of variances of the obtained μmax (Levene’s test, F = 6.02, p = 0.0028), Welch’s ANOVA was used, together with t-tests with non-pooled standard deviations and Holm adjustment for multiple comparisons to maintain a 95% family-wise confidence level.

The lag phase length of the cultures was obtained for all replicates of each strain, and an average lag phase per strain was obtained. Significance of differences between average lag phases was determined as mean ± SD, using a one-way ANOVA test. Tukey’s post hoc test was used to determine significant differences between specific strains. Statistical significance was indicated as p < 0.05.

### Oxidative stress tolerance measurements

The tolerance to oxidative stress was tested by growing the sZJD23 and sZJD28 strains in solid and liquid media and with different concentrations of H_2_O_2_. The spot cultivation was carried out on Delft agar plates with H_2_O_2_ at concentrations of 0.4, 0.6, 0.8, and 1 mM. The strain pre-inocula were grown in Delft liquid media (4 mL) to the stationary phase, and 1 OD_600nm_ of cells was pelleted and spotted as 6 μL droplets in a series of 4 sequential dilutions, 1:5 per plate. The plates were incubated at 30°C for 168 h. For growth assays in liquid cultures, the strains were pre-inoculated and grown in Delft liquid medium (30 mL) to the exponential phase and distributed on 48-well MTP FlowerPlates (m2p-labs) to a starting OD_600nm_ of 0.5. Cell growth was measured for technical quadruplicates under 0, 1, 2, and 3 mM concentrations of H_2_O_2_ using a BioLector reader (m2p-labs) at 30°C and 1000 RPM, and analyzed with the growthrates package in R.

The *μ*_*max*_ of the sZJD23 and sZJD28 strains was calculated in liquid medium under the different H_2_O_2_ concentrations, and the ratios between *μ*_max_ sZJD28 and *μ*_max_ sZJD23 were obtained. The ratios were calculated for all the possible combinations of replicates of both strains per H_2_O_2_ condition. Significance of differences observed between *μ*_*max*_ ratios under the different H_2_O_2_ concentrations was determined as mean ± SD and by using a one-way ANOVA test. Tukey’s post hoc test was used to determine significant differences between specific groups. Statistical significance was indicated as p < 0.05. Additionally, the average *μ*_max_ of each strain was compared using a two-sample t-test in each H_2_O_2_ concentration.

## Supplementary Information

Supplementary Information 1.

Supplementary Information 2.

## Data Availability

The mass spectrometry proteomics data have been deposited to the ProteomeXchange Consortium via the PRIDE partner repository with the dataset identifier PXD074337. Processed quantitative proteomics data that support the findings presented here are available at the GitHub repository: [https://github.com/SysBioChalmers/sce-crabtree-neg-study).

## Code availability

The source code of the analysis done here and the custom scripts for getting and processing ecModels can be found at the GitHub repository: https://github.com/SysBioChalmers/sce-crabtree-neg-study.
