# Supplementary material for: Model-driven analysis reveals oxidative stress adaptation enabling efficient energy utilization in a Crabtree-negative Saccharomyces cerevisiae
Source: Sci Rep. 2026 Jun 21;16:28412. doi: 10.1038/s41598-026-58495-x (PMC13562746; doi:10.1038/s41598-026-58495-x)
Supplement: Supplementary file 1 [file 41598_2026_58495_MOESM2_ESM.pdf]

Supplementary information

Model-driven analysis reveals oxidative stress adaptation enabling efficient energy utilization in a Crabtree-negative *Saccharomyces cerevisiae.*

Albert Tafur Rangel[1,2,3], Andrés Castillo García[1], Carl Malina[1], Verena Siewers[1,2], Eduard J Kerkhoven[1,2,*]

[1] Department of Life Sciences, Chalmers University of Technology, SE-412 96 Gothenburg, Sweden

[2] Novo Nordisk Foundation Center for Biosustainability, Technical University of Denmark, DK-2800 Kgs.

[3] Department of Microbiology, Universidad Popular del Cesar, Valledupar, Colombia.

*Corresponding e-mail: eduardk@chalmers.se

Supplementary Figures

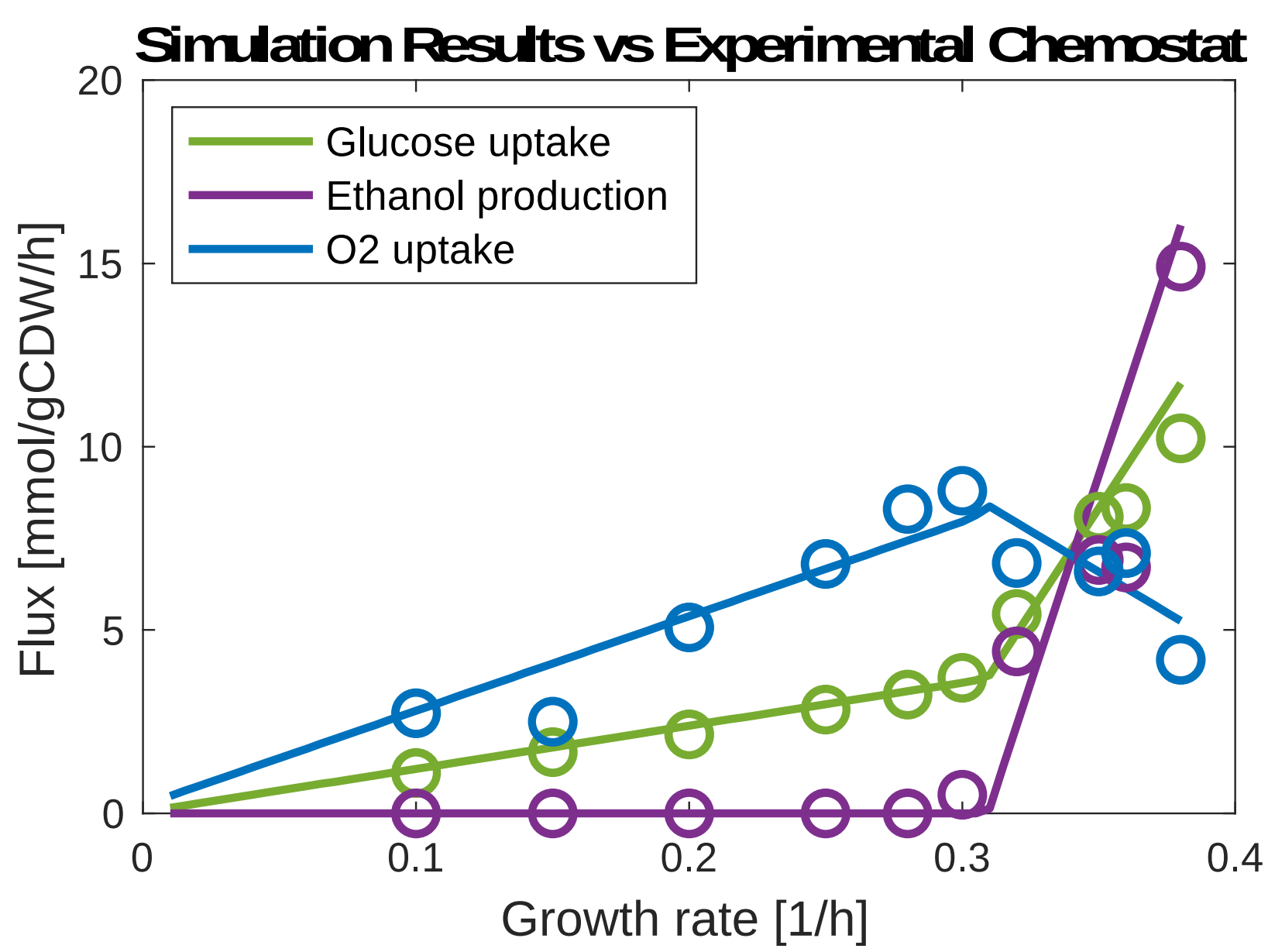

Supplementary Figure 1. Comparison between predicted (lines) and measured (open circles) exchange rates in *S. cerevisiae*. Measured data reported in chemostat.[1]

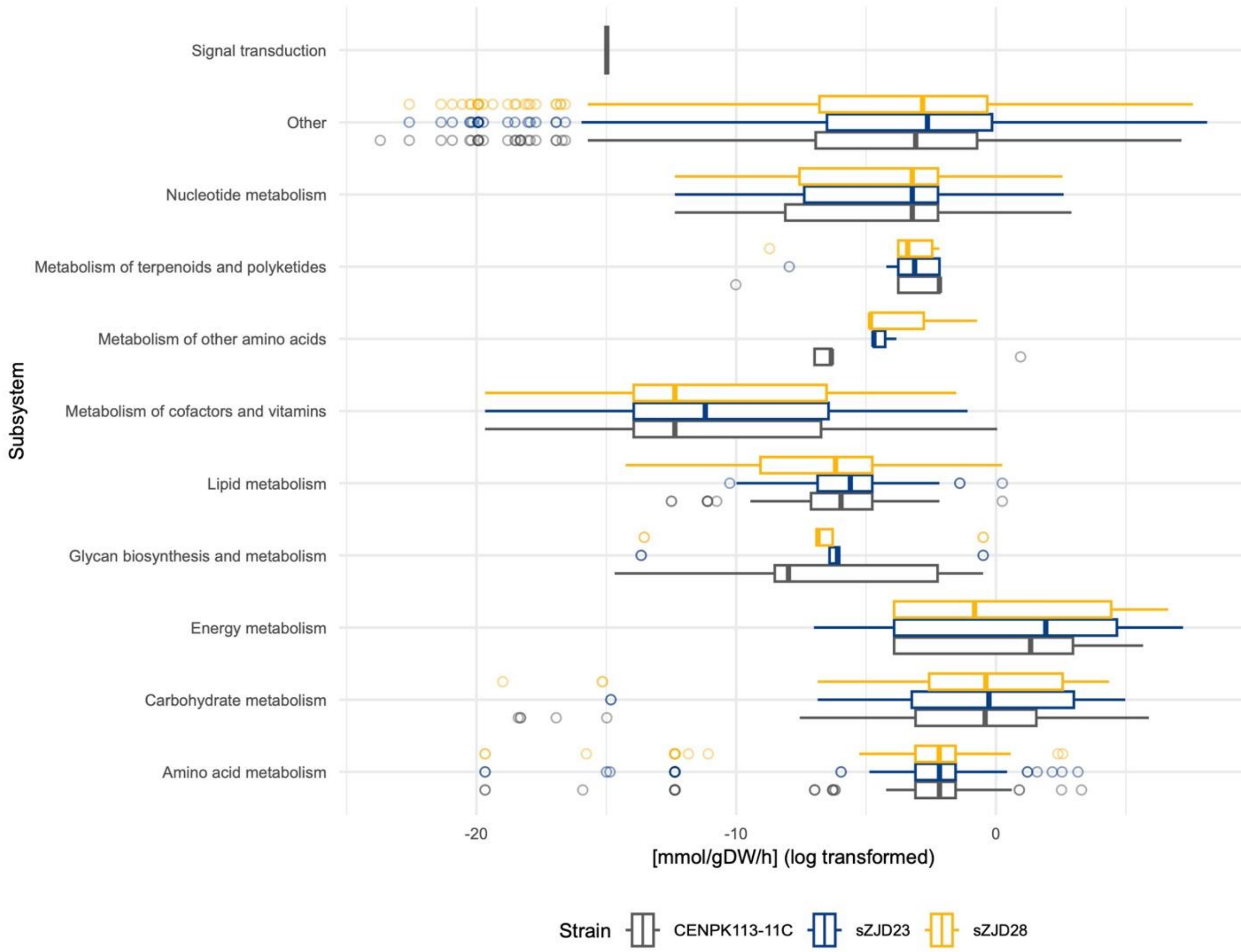

Supplementary Figure 2. Grouped normalized flux distribution by metabolic subsystems in yeast-GEM.

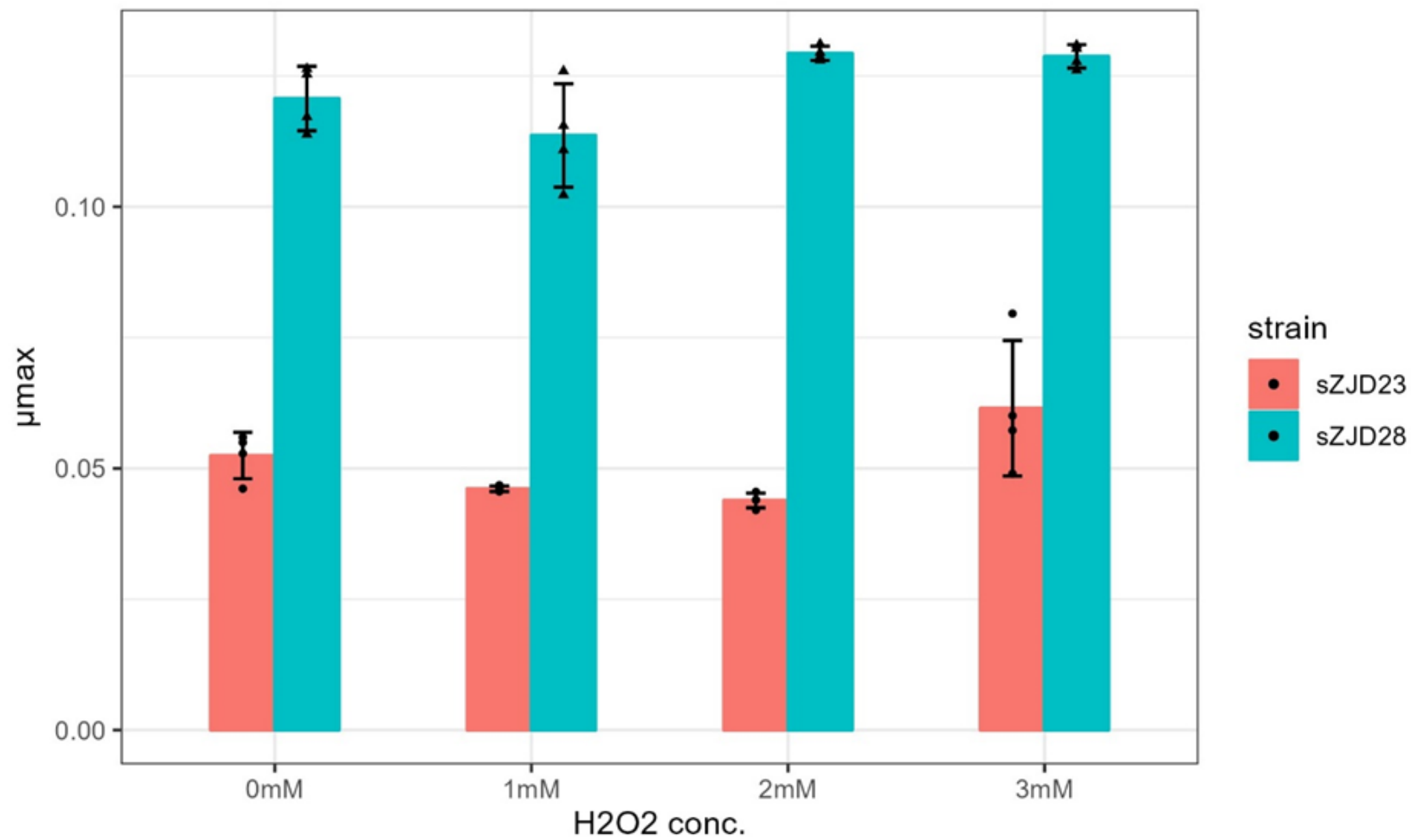

Supplementary Figure 3 average μ_{max} comparison between sZJD28 and sZJD23 in liquid culture under different concentrations of H_2O_2.

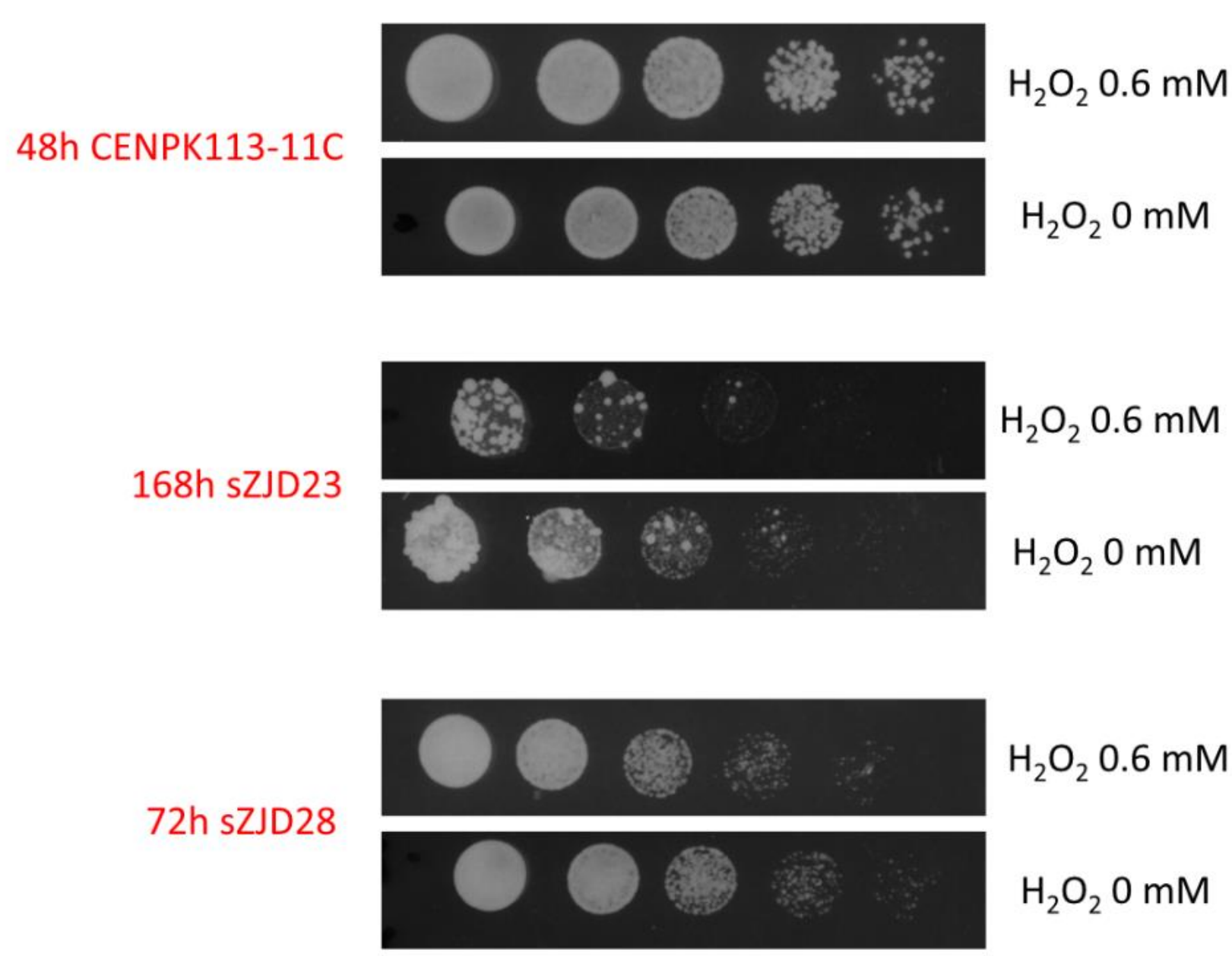

Supplementary Figure 4: Growth in solid media supplemented with H_2O_2 at 0.6 mM concentration. Due to the different growth rates seen in plates for each strain, and to facilitate comparison between them, the images were chosen for the cultivation times when the control plates with no H_2O_2 showed a similar growth for the three strains.

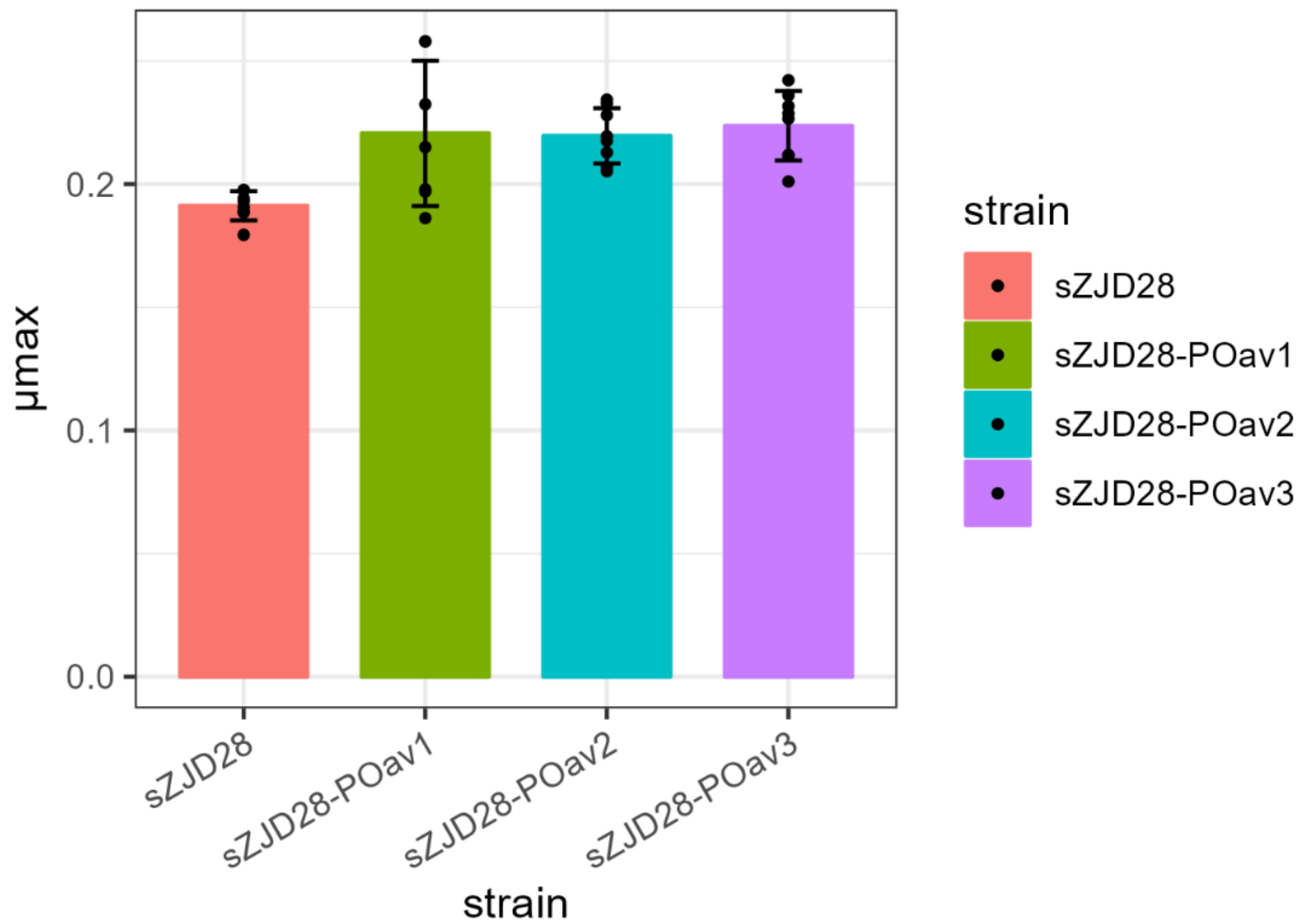

Supplementary Figure 5: Average maximum growth rate comparation between sZJD28 with a second copy of POav integrated.

Supplementary Tables

Supplementary Table 1: Normalized protein usage per KEGG pathway. Values are [g/gDW] / [1/h].

Kegg Pathway	CENPK113_11C	sZJD23	sZJD28
ABC transporters	0.0002	0.0012	0.0007
Alanine, aspartate and glutamate metabolism	0.0087	0.0116	0.0103
Amino sugar and nucleotide sugar metabolism	0.0135	0.0131	0.0136
Aminoacyl-tRNA biosynthesis	0.0185	0.0189	0.0192
Arachidonic acid metabolism	0.0001	0.0007	0.0005
Arginine and proline metabolism	0.0006	0.0016	0.0013
Arginine biosynthesis	0.0060	0.0052	0.0051
Ascorbate and aldarate metabolism	0.0000	0.0000	0.0000
Autophagy - other	0.0018	0.0198	0.0158
Autophagy - yeast	0.0019	0.0201	0.0161
Biosynthesis of unsaturated fatty acids	0.0060	0.0055	0.0051
Biosynthesis of various plant secondary metabolites	0.0036	0.0055	0.0038
Biotin metabolism	0.0001	0.0243	0.0004
Butanoate metabolism	0.0109	0.0103	0.0103
C5-Branched dibasic acid metabolism	0.0032	0.0026	0.0026
Carbapenem biosynthesis	0.0002	0.0002	0.0002
Citrate cycle (TCA cycle)	0.0088	0.0351	0.0250
Cyanoamino acid metabolism	0.0011	0.0007	0.0005
Cysteine and methionine metabolism	0.0141	0.0146	0.0132
D-Amino acid metabolism	NA	0.0002	0.0002
Endocytosis	0.0004	0.0004	0.0004
Ether lipid metabolism	0.0000	0.0000	0.0000
Fatty acid biosynthesis	0.0179	0.0202	0.0202
Fatty acid degradation	0.0010	0.0031	0.0025
Fatty acid elongation	0.0016	0.0011	0.0007
Folate biosynthesis	0.0002	0.0002	0.0002
Fructose and mannose metabolism	0.0327	0.0217	0.0190
Galactose metabolism	0.0136	0.0097	0.0066
Glutathione metabolism	0.0046	0.0049	0.0046
Glycerolipid metabolism	0.0004	0.0027	0.0011
Glycerophospholipid metabolism	0.0091	0.0129	0.0111
Glycine, serine and threonine metabolism	0.0129	0.0144	0.0133
Glycolysis / Gluconeogenesis	0.0981	0.0717	0.0425

Glyoxylate and dicarboxylate metabolism	0.0040	0.0116	0.0080
Histidine metabolism	0.0003	0.0003	0.0003
Inositol phosphate metabolism	0.0016	0.0070	0.0056
Lipoic acid metabolism	0.0061	0.0229	0.0168
Longevity regulating pathway - multiple species	NA	0.0000	0.0000
Lysine metabolism	0.0132	0.0209	0.0173
MAPK signaling pathway - yeast	0.0036	0.0037	0.0037
Mannose type O-glycan biosynthesis	0.0005	0.0005	0.0005
Meiosis - yeast	0.0020	NA	0.0018
Methane metabolism	0.0334	0.0197	0.0138
Monobactam biosynthesis	0.0000	0.0001	0.0000
N-Glycan biosynthesis	0.0004	0.0012	0.0010
Nicotinate and nicotinamide metabolism	0.0013	0.0014	0.0013
Nitrogen metabolism	0.0028	0.0018	0.0016
One carbon pool by folate	0.0070	0.0071	0.0065
Other	0.1271	0.1859	0.1251
Other types of O-glycan biosynthesis	0.0005	0.0005	0.0005
Oxidative phosphorylation	0.0461	0.1091	0.1007
Pantothenate and CoA biosynthesis	0.0040	0.0034	0.0034
Pentose and glucuronate interconversions	0.0005	0.0011	0.0009
Pentose phosphate pathway	0.0269	0.0130	0.0095
Peroxisome	0.0008	0.0051	0.0043
Phagosome	0.0005	0.0060	0.0048
Phenylalanine metabolism	0.0000	0.0002	0.0000
Phenylalanine, tyrosine and tryptophan biosynthesis	0.0052	0.0054	0.0052
Phosphatidylinositol signaling system	0.0011	0.0077	0.0058
Porphyrin metabolism	0.0056	0.0056	0.0056
Propanoate metabolism	0.0056	0.0113	0.0128
Protein export	NA	0.0000	0.0000
Protein processing in endoplasmic reticulum	0.0001	0.0003	0.0003
Purine metabolism	0.0171	0.0204	0.0179
Pyrimidine metabolism	0.0018	0.0028	0.0028
Pyruvate metabolism	0.0289	0.0486	0.0471
RNA degradation	0.0138	0.0075	0.0048
Riboflavin metabolism	0.0006	0.0006	0.0006
Ribosome biogenesis in eukaryotes	0.0000	0.0000	0.0000
Selenocompound metabolism	0.0062	0.0065	0.0077
Sesquiterpenoid and triterpenoid biosynthesis	0.0003	0.0003	0.0003

Starch and sucrose metabolism	0.0072	0.0081	0.0066
Steroid biosynthesis	0.0101	0.0095	0.0095
Sulfur metabolism	0.0012	0.0005	0.0007
Sulfur relay system	0.0004	0.0003	0.0003
Terpenoid backbone biosynthesis	0.0116	0.0121	0.0118
Thiamine metabolism	0.0011	0.0011	0.0010
Tryptophan metabolism	0.0029	0.0086	0.0066
Tyrosine metabolism	0.0005	0.0005	0.0000
Ubiquinone and other terpenoid-quinone biosynthesis	0.0000	0.0000	0.0000
Valine, leucine and isoleucine metabolism	0.0195	0.0219	0.0197
Various types of N-glycan biosynthesis	0.0003	0.0009	0.0007
Vitamin B6 metabolism	0.0075	0.0050	0.0047
beta-Alanine metabolism	0.0000	0.0000	0.0000

Supplementary Table 2: Protein with a capacity usage of more than 0.75 after proteomics integration to an ecModel

Proteins with a capacity usage of more than 0.75	count	Intersection
ACC1, ARG8, TRP3, ERG27, FBA1, PMT4, TDH1, SHM1, HXK2, BNA2, MET16, PFK1, PFK2, PRS3, PDX3, RKI1, ERG6, TRR1, YPQ1, SAM3, FAS1, FAS2, ZRC1	23	CENPK113 11C
DPS1, COX13, PRO1, GLN4, KGD2, LSC2, SDH2, SDH1, PIC2, TAM41, ATM1	11	sZJD23
SPXB, FAA2, LYS20, LSC1, MVD1, BNA6, ERG1, DIP5, AGP2, PTR2	10	sZJD28
ILV6, CIT3, ARG5,6, ATP1, ATP2, ATP18, COX5A, HXK1, NDE1, THS1, TPO4, PNS1	12	CENPK113 11C..sZJD23
KRE6, ADE6, ERG20, QCR7, RIP1, GLK1, BNA4, ALT1, MCT1, unknown, TKL2, HXT2, HXT3, LYP1	14	CENPK113 11C..sZJD28
GSC2, ATP17, COX2, KGD1, HMG2, IDP2, GND2, PDB1, LAT1, PYK2, VBA1, GPT2, IRC24, LIP5, ALG5, HPA3	16	sZJD23..sZJD28
FKS1, PUT2, ILV2, LEU1, LYS4, MRI1, ACH1, PDH1, ADK1, ADE13, TRP4, ASN2, ATP3, ATP5, ATP7, ATP4, ATP20, BIO2, ERG25, ERG4, ARO2, CYS3, URA4, PMT1, COR1, QCR10, QCR2, MIS1, GUS1, TDH3, GUT2, LPD1, GRS1, GUA1, LYS21, ERG13, ADE17, IDI1, LAP2, SAM2, MES1, QNS1, NDI1, URA5, PCM1, SEC53, SER1, PDA1, PDX1, YCF1, RNR1, SES1, TRP5, QRI1, UGP1, PUS1, ODC2, FCY2, AAC3, YHM2, CAN1, AVT3, MUP1, OAC1, PHO91, MIR1, YMC1, TSC13, OLE1, FAA1, FAA4, CDS1, PSD1, NEO1, NFS1, ADP1, SPF1, COT1, DUG1, STT3, FRE1, ALG9, ALG12, CTS1, ZRT1	85	CENPK113 11C..sZJD23..sZJD28

Supplementary Table 3: average μ_{max} of strains and standard deviation, statistically compared per H_2O_2 concentration in liquid cultures, and μ_{max} ratio between sZJD28 and sZJD23.

H2O2 concentration (mM)	Strain	μ_{max} mean	μ_{max} SD	P value (t-test)	μ_{max}-sZJD28 / μ_{max}-sZJD23
0	sZJD23	0,052474853	0,004423012	4.523e-06	2,300
0	sZJD28	0,120676419	0,006137552		
1	sZJD23	0,046109393	0,000514918	0.0008229	2,464
1	sZJD28	0,113632897	0,009863452		
2	sZJD23	0,043883746	0,001425426	1.631e-10	2,947
2	sZJD28	0,129318663	0,001340459		
3	sZJD23	0,061480499	0,012956409	0.001549	2,094
3	sZJD28	0,128733391	0,002219116		

Supplementary Table 4: list of primers used in this study.

Primer	Sequence	Purpose
1	TTGGTATGTTTATTCGTATAAGTTAC	amplification of homologous site X3-UP for integration
2	TAAAAAAGGAGTAGAAACATTTTGAAGCTATGGTGTGTGCAGTCTCGTATGTCGGCTCTC	
3	ACAATAGGCAAGAAGTAGGCGAGAGCCGACATACGAGACTGCACACACCATAGCTTCAAA	amplification of pTEF1+PO cassette
4	CTTATTTAATAATAAAAATCATAAATCATAAGAAATTCGCTTACTTAATATATTTAGATTCTAAACCT	
5	CTTGGAAGCAGAAGGTTTAGAATCTAAATATATTAAGTAAGCGAATTTCTTATGATTTATGATTTT	amplification of tADH1
6	CTTTTACTAGCATATCAATATCCGTTTCATTGAAAAGTGGGAGCGACCTCATGCTATAC	
7	TAGGTCAGGTTGCTTTCTCAGGTATAGCATGAGGTCGCTCCCACTTTTCAATGAAACGGA	amplification of homologous site X3-DOWN for integration
8	GAGGTGGTTATTGATCACCG	
9	TATAGACCCAGCTATGTTAG	check for integration of PO expression cassette
10	CCGTGCAATACCAAAATCG	
11	GCAGTGAAAGATAAATGATCCTAATGTGTCCGCGTTTCTAGTTTTAGAGCTAGAAATAGC	For introduction of gRNA targeting X-3 in the plasmid pMEL10 and PCR amplification of it
12	GATCATTTATCTTTCACTGCGGAG	

Supplementary Table 5: list of plasmids used in this study.

Plasmid list
pMC-X3
pMEL10
pYTK053

Sequence of the POav integration cassette:

TTGGTATGTTTATTCGTATAAGTTACTGTTGTCCACAGGCAATACTCTGCAGAAAATTAAAA
CGGCATTAATGCTAGGACAACCAGAATTGTTACTACTGTATGTGCGATAGTTGATAACTGCA
ACATTATGCCCGGTATATTCTCAAAAAACCCTATTACTGCATACGAAGAAATCGCAAGAGAA
ATCTTTCGGTTTGGAAAAGCTCACTGTGAGGTTCCTTGGAGCCAATAGTAATACAGCACAA
TCCAAGGAAAAATCTGGCCTATATGCAAGGAAGGAGAGATAGTCAAAAGCATTCTTTCCCC
TAGAAGTTGGTGCATATATGGCATCGTTAAAACATATTACCCCCAAAATTTCTTCTCTAAAC
GATGTGCTTGGCCTTTGTTTTGGTTTTTGATGTCGGTCGTTTGAGGCCCCTTGCGGAAAAT
CGAGATCGCCGAATGGCACGCGAGGGAAGGGAAATAAGGTTTAAAGGCACTGAAACAATA
GGCAAGAAGTAGGCGAGAGCCGACATACGAGACTGCACACACCATAGCTTCAAAATGTTT
CTACTCCTTTTTTACTCTTCCAGATTTTCTCGGACTCCGCGCATCGCCGTACCACTTCAAAA
CACCCAAGCACAGCATACTAAATTTCCCCTCTTTCTTCCTCTAGGGTGTCGTTAATTACCCG
TACTAAAGGTTTGGAAAAGAAAAAAGAGACCGCCTCGTTTCTTTTTCTTCGTCGAAAAAGGC
AATAAAAATTTTTATCACGTTTCTTTTTCTTGAAAATTTTTTTTTTTTGATTTTTTTCTCTTTCGA
TGACCTCCCATTGATATTTAAGTTAATAAACGGTCTTCAATTTCTCAAGTTTCAGTTTCATTT
TTCTTGTTCTATTACAACTTTTTTTACTTCTTGCTCATTAGAAAGAAAGCATAGCAATCTAATC
TAAGTTTTAATTACAAATGTCCGACAACAAGATCAACATTGGTTTGGCAGTTATGAAAATATT
GGAATCCTGGGGTGCTGACACTATCTACGGTATCCCTTCAGGTACTTTGTCTTCATTAATG
GATGCTATGGGTGAAGAAGAAAACAACGTAAAGTTCTTGCAAGTTAAGCATGAAGAAGTCG
GTGCAATGGCTGCAGTTATGCAATCCAAATTCGGTGGTAATTTGGGTGTAACAGTTGGTTC
CGGTGGTCCAGGTGCTAGTCACTTGATTAACGGTTTATACGATGCCGCTATGGACAATATT
CCAGTTGTCGCTATATTGGGTTCAAGACCTCAAAGAGAATTGAACATGGATGCATTCCAAG
AATTGAACCAAAACCCAATGTACGATCATATTGCCGTATACAATAGAAGAGTTGCCTATGCT
GAACAATTGCCTAAATTAGTTGATGAAGCAGCCAGAATGGCAATCGCCAAGAGAGGTGTCG

CCGTATTAGAAGTCCCTGGTGACTTCGCTAAGGTAGAAATTGATAACGACCAATGGTACTC
CAGTGCTAATTCCTTGAGAAAATATGCACCAATAGCCCCTGCTGCACAAGATATCGACGCC
GCTGTAGAATTGTTGAACAACAGTAAGAGACCTGTTATCTACGCAGGTATTGGTACTATGG
GTCACGGTCCAGCCGTCCAAGAATTAGCTAGAAAAATCAAGGCACCTGTAATTACTACAGG
TAAAAATTTCGAAACATTCGAATGGGATTTTGAAGCATTGACAGGTTCAACCTATAGAGTTG
GTTGGAAACCAGCCAACGAAACTATTTTGGAAGCAGATACAGTCTTATTCGCCGGTTCTAA
TTTTCCTTTCTCAGAAGTTGAAGGTACTTTTAGAAACGTCGATAACTTCATCCAAATAGATAT
AGACCCAGCTATGTTAGGTAAAAGACATCACGCTGATGTTGCAATTTTGGGTGACGCCGGT
TTGGCTATCGATGAAATCTTGAATAAGGTTGATGCTGTCGAAGAATCTGCATGGTGGACAG
CCAATTTGAAGAACATTGCAAACTGGAGAGAATACATCAACATGTTGGAAACCAAGGAAGA
GGGTGACTTGCAATTCTACCAAGTTTACAACGCTATAAATAACCATGCTGATGAAGACGCA
ATCTATTCTATAGATGTTGGTAACTCTACCCAAACTTCAATTAGACATTTGCACATGACCCC
AAAGAATATGTGGAGAACCTCACCTTTATTTGCCACTATGGGTATCGCTATTCCAGGTGGTT
TGGGTGCTAAAAACACTTATCCTGACAGACAAGTTTGGAATATTATAGGTGACGGTGCATT
CTCTATGACATACCCAGACGTAGTTACCAACGTTAGATATAATATGCCTGTTATCAACGTCG
TATTTTCAAACACAGAATACGCTTTCATTAAAAATAAGTATGAAGATACCAATAAGAACTTGT
TTGGTGTCGACTTCACTGATGTAGACTACGCTAAAATAGCTGAAGCACAAGGTGCAAAGGG
TTTTACCGTTTCTAGAATCGAAGATATGGACAGAGTTATGGCCGAAGCTGTCGCAGCCAAT
AAGGCTGGTCATACTGTTGTCATAGACTGTAAGATCACACAAGATAGACCAATTCCTGTTGA
AACATTGAAGTTGGACTCCAAGTTGTACAGTGAAGATGAAATTAAAGCTTACAAGGAAAGAT
ACGAAGCTGCAAATTTGGTTCCTTTTAGAGAATACTTGGAAGCAGAAGGTTTAGAATCTAAA
TATATTAAGTAAGCGAATTTCTTATGATTTATGATTTTTATTATTAAATAAGTTATAAAAAAAAT
AAGTGTATACAAATTTTAAAGTGACTCTTAGGTTTTAAAACGAAAATTCTTATTCTTGAGTAA
CTCTTTCCTGTAGGTCAGGTTGCTTTCTCAGGTATAGCATGAGGTCGCTCCCACTTTTCAAT
GAAACGGATATTGATATGCTAGTAAAAGGACGAGCTCAAGAGCGAAAATATAAGTAAAGAA
TTCGAGTGCACTTGTCTCCATGCAGCAAGATTTCATATGAGTCTTTTTTATCTTTTTACTTTT
TACATTACACGATATGCACTTTATGAAAATTTAACGAGGTTGGAAGCCGGATAATCAACCAA
AATCAGGCACGAAGGCACACTCGTATATGCATGTTGTTGAAACTCTGTTACGCTGAACTAA
CAATCACACATGTAGAGGTCACCGGGAAAAGTTGCGACCCCATGGAAGGTCGATCTCTTC
GTTTGGCTTTGCTTGGCTGGCGGCATTGCGCTTCTTCGCTTATACCCGACTCTTGACGCTC
GAGCTCGTTCATTGAGATACCTTTATTCTTGCACATTTTCTGGCTTTTTTCGCTACTCGGGT
ACATGTAATCATGCACACAGAAGGTGCTGTAGGGTGAAAGTTCCTTTGTGCTGTCGTTTGT
TTTTAATGCCAAACTTTCCGGTGATCAATAACCACCTC

Supplementary References 1

1. Chen, Y. & Nielsen, J. Energy metabolism controls phenotypes by protein efficiency and allocation. *Proc Natl Acad Sci U S A* **116**, 17592–17597 (2019).
